# *Bacillus anthracis* Spore Surface Protein BclA Mediates Complement Factor H Binding to Spores and Promotes Spore Persistence

**DOI:** 10.1371/journal.ppat.1005678

**Published:** 2016-06-15

**Authors:** Yanyu Wang, Sarah A. Jenkins, Chunfang Gu, Ankita Shree, Margarita Martinez-Moczygemba, Jennifer Herold, Marina Botto, Rick A. Wetsel, Yi Xu

**Affiliations:** 1 Center for Infectious and Inflammatory Diseases, Institute of Biosciences and Technology, Texas A&M Health Science Center, Houston, Texas, United States of America; 2 Department of Microbial Pathogenesis and Immunology, College of Medicine, Texas A&M Health Science Center, College Station, Texas, United States of America; 3 Department of Medicine, Imperial College London, London, United Kingdom; 4 The Brown Foundation Institute of Molecular Medicine for the Prevention of Human Diseases, University of Texas Health Science Center, Houston, Texas, United States of America; The University of Texas-Houston Medical School, UNITED STATES

## Abstract

Spores of *Bacillus anthracis*, the causative agent of anthrax, are known to persist in the host lungs for prolonged periods of time, however the underlying mechanism is poorly understood. In this study, we demonstrated that BclA, a major surface protein of *B*. *anthracis* spores, mediated direct binding of complement factor H (CFH) to spores. The surface bound CFH retained its regulatory cofactor activity resulting in C3 degradation and inhibition of downstream complement activation. By comparing results from wild type C57BL/6 mice and complement deficient mice, we further showed that BclA significantly contributed to spore persistence in the mouse lungs and dampened antibody responses to spores in a complement C3-dependent manner. In addition, prior exposure to BclA deletion spores (Δ*bclA*) provided significant protection against lethal challenges by *B*. *anthracis*, whereas the isogenic parent spores did not, indicating that BclA may also impair protective immunity. These results describe for the first time an immune inhibition mechanism of *B*. *anthracis* mediated by BclA and CFH that promotes spore persistence *in vivo*. The findings also suggested an important role of complement in persistent infections and thus have broad implications.

## Author Summary

We discovered an immune modulatory mechanism of *Bacillus anthracis* mediated by the spore surface protein BclA. We showed for the first time that BclA mediated the binding of complement factor H, a major negative regulator of complement, to the surface of spores. The binding led to the down-regulation of complement activities *in vitro* and in an animal model. Using mice deficient in complement components, we further showed that BclA promoted spore persistence in the mouse lungs and impaired antibody responses against spores in a complement-dependent manner. We further provided evidence suggesting a role of BclA in the development of protective immunity against lethal *B*. *anthracis* challenges. These findings draw attention to a previously understudied aspect of the complement system. They suggest that in addition to conferring resistance to complement-mediated killing and phagocytosis, complement inhibition by pathogens have long-term consequences with respect to persistent infections and development of protective immunity. Considering a growing list of microbial pathogens capable of modulating complement activities, our findings have broad implications.

## Introduction

Persistent colonization of the host by microbial pathogens can cause chronic infections, which are often difficult to treat with conventional antibiotics. It is recognized that persistent infection is a unique phase often involving specific virulence factors and pathogenic mechanisms [1]. Identifying and understanding these persistent mechanisms is key to developing new strategies to more effectively combat chronic infections.

*Bacillus anthracis* is a spore forming, Gram-positive bacterium that causes anthrax. Infections are initiated by entry of spores into the host via the respiratory system, the gastrointestinal tract, or cuts/wounds in the skin. Among the three forms of anthrax infections, inhalational anthrax has the highest mortality rate. One of the characteristic features of inhalational anthrax is the ability of spores to persist in the host lungs for prolonged periods of time [2–7]. Viable spores can be recovered from the lungs of exposed animals including non-human primates weeks or even months after the initial exposure. In addition, incubation periods of up to 43 days have been observed in humans [6]. This led to the 60-day antibiotic regimen recommended by the Centers for Disease Control and Prevention for people with pulmonary exposure to *B*. *anthracis* spores [7].

The mechanism underlying *B*. *anthracis* spore persistence is poorly understood. Mechanisms used by other bacterial pathogens for persistent infections include biofilm formation [8–12], residing in intracellular niches [13–15], suppression of innate and adaptive immune responses [13, 16–18], and changes in bacterial physiology and metabolism that favor persistent colonization [19–21]. *B*. *anthracis* spores are metabolically inactive and resistant to microbicidal effectors present *in vivo*. It was originally thought that the dormancy and resilience of spores were responsible for their ability to persist in the host. However, in a mouse model for spore persistence, *B*. *anthracis* spores were found to be significantly better at persisting in the lungs than *Bacillus subtilis* spores, suggesting the existence of persistence-promoting mechanism(s) beyond spore dormancy and resilience [4]. *B*. *anthracis* spores were also observed to be distributed throughout the lungs as single spores with the majority being extracellularly located [4], suggesting that biofilm formation or hiding in an intracellular niche is unlikely to be the major underlying mechanism. It is known that pulmonary exposure to *B*. *anthracis* spores does not elicit robust inflammatory immune responses in the lungs. Although the spore surface lacks typical pathogen-associated molecular patterns such as lipopolysaccharides, lipotechoic acid, and flagellin [22], spores have been shown to be capable of activating Toll-like receptor 2 and MyD88-dependent signaling [23], triggering inflammatory cytokine production [24, 25], and activating natural killer cells [26, 27]. Therefore the subdued immune response is likely due to an active immune evasion/suppression mechanism rather than a passive inactivity of the spores. The anthrax toxins are known to inhibit host immune responses. However, spores of a *B*. *anthracis* strain devoid of the anthrax toxins persisted as well as the parent toxin-producing strain [4]. This speaks against the possibility that low levels of anthrax toxins produced by a small amount of germinated spores *in vivo* may inhibit the overall immune response in the lungs and contribute to spore persistence. These observations provide support for a spore-mediated mechanism of immune suppression that has yet to be identified.

*Bacillus* collagen-like protein of *anthracis* (BclA) is the most abundant protein on the exosporium, the outermost layer of *B*. *anthracis* spores. It is the structural component of the hair-like nap on the exosporium [28]. Because of this spatial localization, BclA sits at the forefront with respect to interactions with host factors upon entry into the host. A number of studies have shown that BclA mediates spore uptake by macrophages and epithelial cells in both complement-dependent and–independent manners [29–33]. However despite its abundance, localization and interactions with host cells, the precise role of BclA in *B*. *anthracis* pathogenesis remains unclear. In animal models of acute anthrax infections BclA did not appear to contribute to virulence [29, 34].

In this study, the ability of BclA to manipulate the complement system and its role in spore survival and persistence *in vivo* was investigated. We found that BclA mediated the recruitment of complement factor H (CFH), the major inhibitor of the alternative pathway, to the spore surface where it facilitated C3 degradation; thereby inhibiting downstream complement activation. We further showed that BclA significantly promoted spore persistence in the mouse lungs and dampened antibody responses to spores in a complement-dependent manner. Finally we showed that BclA impaired protective immunity against lethal *B*. *anthracis* challenges. These findings have important implications in *B*. *anthracis* pathogenesis, bacterial manipulation of complement and persistent infections in general.

## Results

### The *B*. *anthracis* collagen-like protein BclA mediated CFH binding to spores

**Fig 1:**
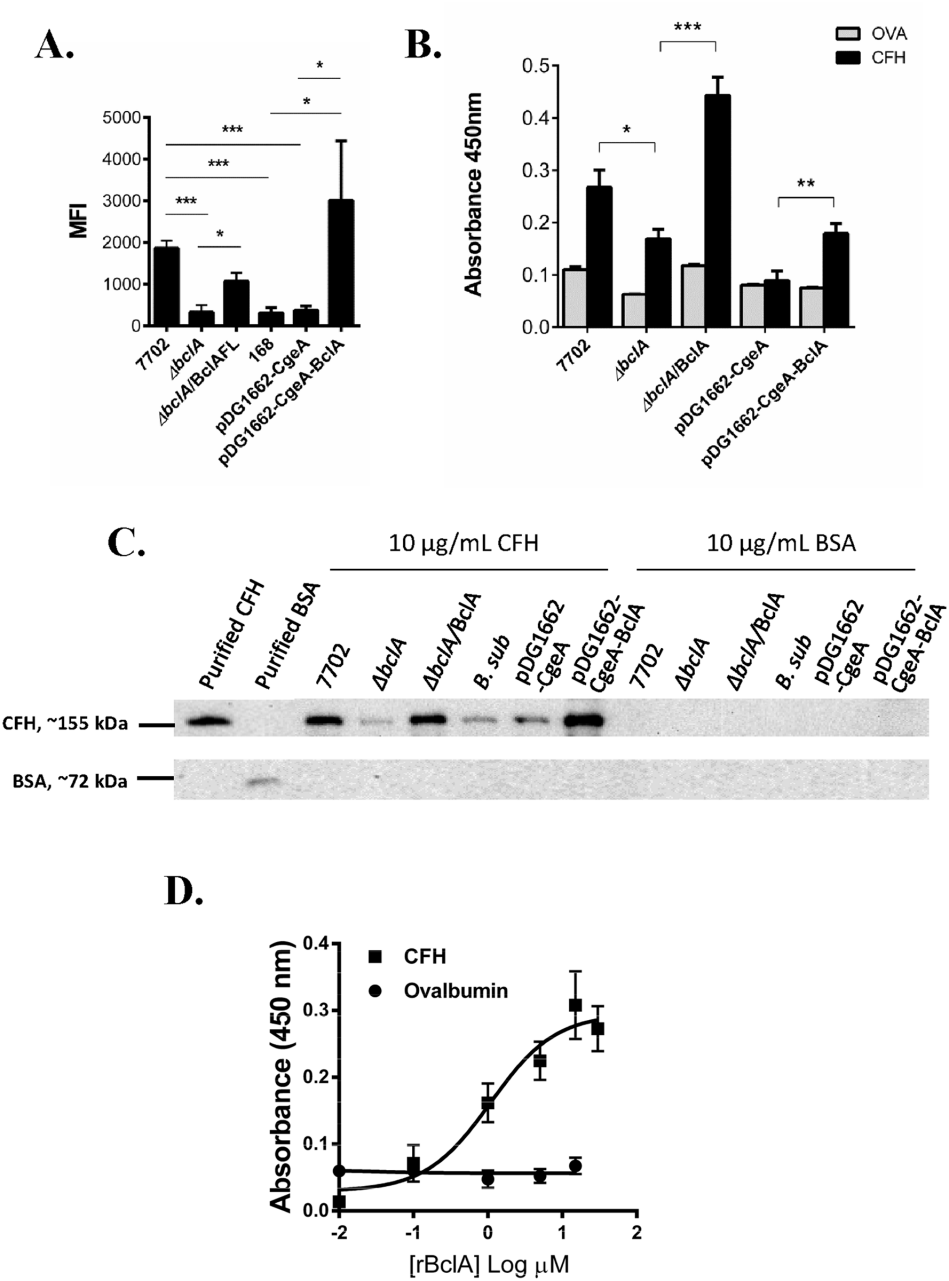
*B*. *anthracis* spore surface protein BclA mediated CFH binding to spores. Spores were incubated with purified human CFH in PBS buffer containing D-alanine. Spore-bound CFH was determined by flow cytometry (**A**), solid phase binding assay (**B**) and Western blot (**C**). Flow cytometry results were combined from at least three independent experiments. Solid phase binding assay results were combined from two independent experiments, each with duplicate wells. Western blots shown were representative of at least three independently performed experiments. (**D**) Recombinant BclA protein (rBclA) bound to immobilized human CFH in a concentration-dependent manner. Results were combined from three independent experiments. *, *p* < 0.05; **, *p* < 0.01; ***, *p* < 0.001; *t* test.

Spores of *B*. *anthracis* Sterne strain 7702 and the isogenic BclA deletion mutant (Δ*bclA*) were incubated with purified human CFH. Spore-CFH interaction was analyzed using flow cytometry (Fig 1A), solid phase binding assays (Fig 1B) and spore pull down assays (Fig 1C). In all three different assays, deletion of BclA led to significantly reduced CFH binding compared to 7702 spores. Complementation of the deletion with the full-length *bclA* gene (Δ*bclA*/BclA) restored CFH binding (Fig 1A–1C). Surface expression of BclA in the complemented strain was confirmed by immunofluorescence microscopy and flow cytometry (S1 Fig).

We next investigated if BclA could mediate recruitment of CFH from human and mouse serum, and mouse bronchial alveolar lavage (BAL) fluids. In order to distinguish between direct CFH binding and indirect binding through C3 fragments deposited on the spore surface, the binding assays were performed using heat-treated serum and BAL fluids so that the complement system was inactivated while CFH remained functional [35]. 7702 spores were able to recruit more CFH from normal human serum (NHS), mouse serum and mouse BAL fluids, compared to Δ*bclA* and *B*. *subtilis* spores, respectively (S2 Fig).

To further determine if BclA was sufficient to mediate CFH binding to spores, we expressed BclA on the surface of *B*. *subtilis* spores, which do not contain any BclA-encoding genes. Surface expression was verified by immunofluorescence microscopy and flow cytometry (S1 Fig). We observed that expression of BclA significantly enhanced the binding of purified CFH and CFH in human serum, mouse serum and mouse BAL fluids to *B*. *subtilis* spores (Fig 1A–1C and S2 Fig). BclA was further expressed as a His-tag recombinant protein (rBclA). Results from ELISAs showed that rBclA bound to CFH in a concentration-dependent and saturable manner, with an apparent K_*D*_ of 0.91±0.45 μM (Fig 1D). Taken together, the results described above indicated that *B*. *anthracis* spore surface protein BclA mediated direct binding of human and mouse CFH to spores.

### BclA-mediated CFH binding promoted degradation of C3b to iC3b and inhibited further C3 activation

**Fig 2:**
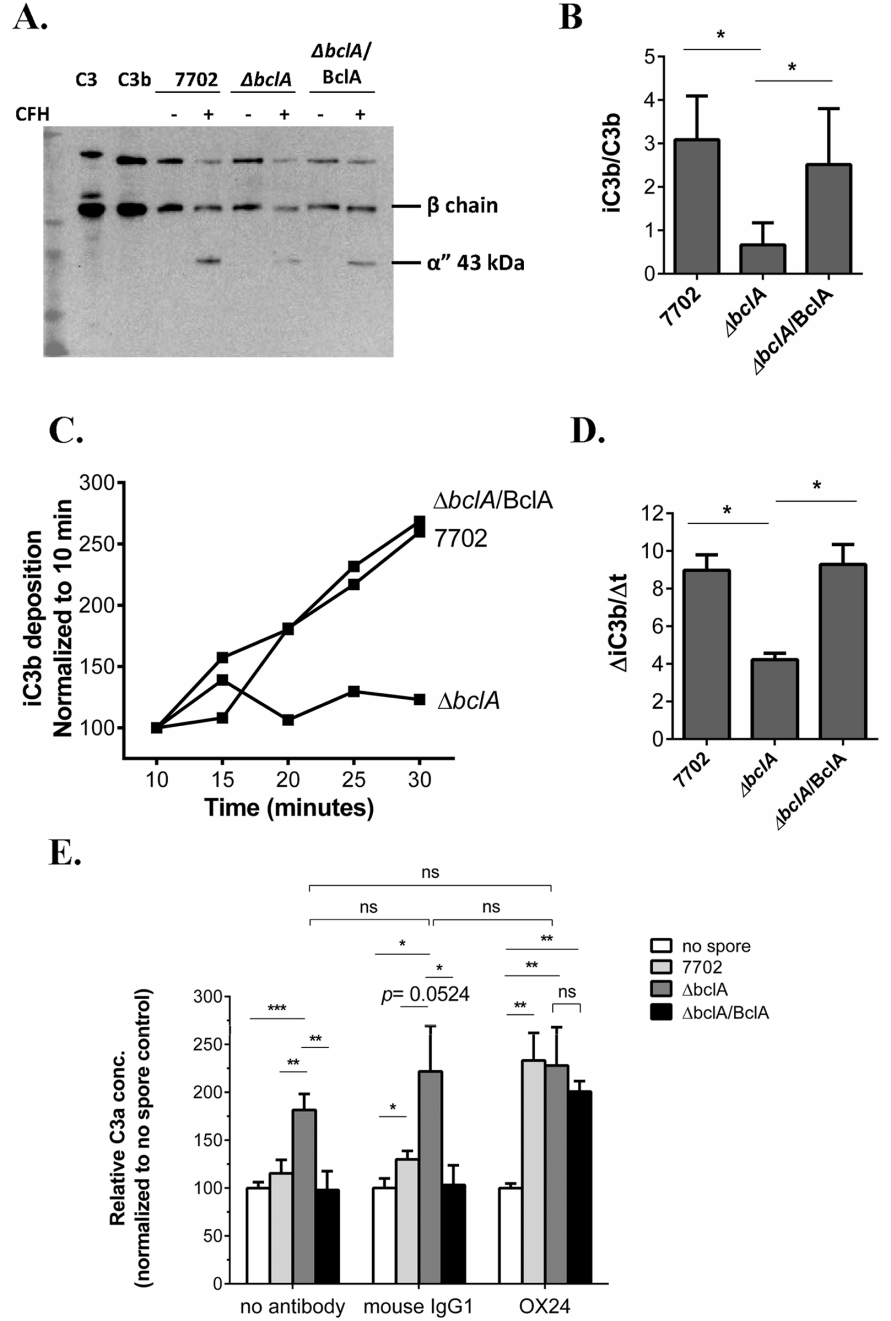
BclA-mediated CFH binding promoted degradation of C3b on the spore surface and downregulated further C3 activation. (**A**) and (**B**) Spores were incubated with purified human C3b, CFH and CFI. C3 fragments deposited on the spore surface were detected using anti-C3 polyclonal antibodies (**A**). The image shown is representative of at least three independent experiments. The ratio of iC3b/C3b was determined by quantifying the density of the corresponding bands in western blots using Image J (**B**). The β chain represents C3b + iC3b, and the α” chain represents iC3b. Results were combined from three independent experiments. (**C**) and (**D**) Rate of iC3b deposition on spores. Spores were incubated with 10% NHS for the indicated time and subjected to flow cytometry analysis using iC3b-specific antibody. The results shown are mean fluorescence intensity normalized to that at 10 minutes, respectively, and combined from at least three independent experiments (**C**). The rate of iC3b deposition (ΔiC3b/Δt) was calculated by linear regression analysis of the normalized data (GraphPad Prism 6) (**D**). (**E**) Determination of C3a concentration. GVB^0^ buffer containing 20% NHS (no antibody), 20% NHS pre-treated with the CFH functional blocking antibody OX24 (240 nM final conc.) or mouse IgG1 control (240 nM) was incubated with no spore, 7702, Δ*bclA* or Δ*bclA*/BclA spores at 37°C for 30 min and centrifuged to remove the spores. C3a concentrations in the supernatants were determined using the Human C3a ELISA kit (BD OptEIA^™^) and normalized to the respective no spore control. Data was combined from four experiments, each with duplicate wells. *, *p* < 0.05; **, *p* < 0.01; ***, *p* < 0.001; *t* test.

One of the principal functions of CFH is to act as a co-factor for complement factor I (CFI) to cleave C3b to the inactive iC3b, which disrupts the formation of the alternative complement pathway (ACP) C3 convertase. We first investigated the effect of BclA-mediated CFH recruitment on C3b cleavage to iC3b on the spore surface using purified complement components C3b, CFI and CFH. The results showed that the iC3b/C3b ratio on Δ*bclA* spores was significantly lower than that on 7702 and Δ*bclA*/BclA spores (Fig 2A and 2B). We further incubated the different spores with NHS for various length of time. The rate of iC3b accumulation on Δ*bclA* spores was significantly slower compared to that on 7702 and Δ*bclA*/BclA spores (Fig 2C and 2D). These results indicated that BclA-mediated CFH recruitment significantly promoted the cleavage of C3b to iC3b on the spore surface.

The increased cleavage of C3b to iC3b in the presence of BclA could potentially reduce the available C3b necessary for efficient C3 convertase formation, thereby reducing further C3 activation. We therefore determined if BclA-mediated CFH recruitment affected C3a production in NHS incubated with the different spores. The results showed that C3a concentration was significantly higher in samples incubated with Δ*bclA* spores compared to those incubated with 7702 or Δ*bclA*/BclA spores (Fig 2E, no antibody), suggesting that C3 cleavage was inhibited in the presence of BclA-expressing spores. To further determine whether the inhibition was due to CFH, we tested the effect of a CFH functional blocking antibody (OX24) [36]. Pre-treatment of NHS with OX24 increased the C3a concentration in samples incubated with 7702 or Δ*bclA*/BclA spores to a similar level as that seen in those with Δ*bclA* spores; whereas pre-treatment with the isotype control antibody (mouse IgG1) showed a similar pattern as that seen in the no antibody control (Fig 2E). Taken together, these results suggested that BclA-mediated CFH recruitment significantly reduced further activation of C3.

### BclA-mediated CFH recruitment led to inhibition of downstream complement activation *in vitro* and *in vivo*

**Fig 3:**
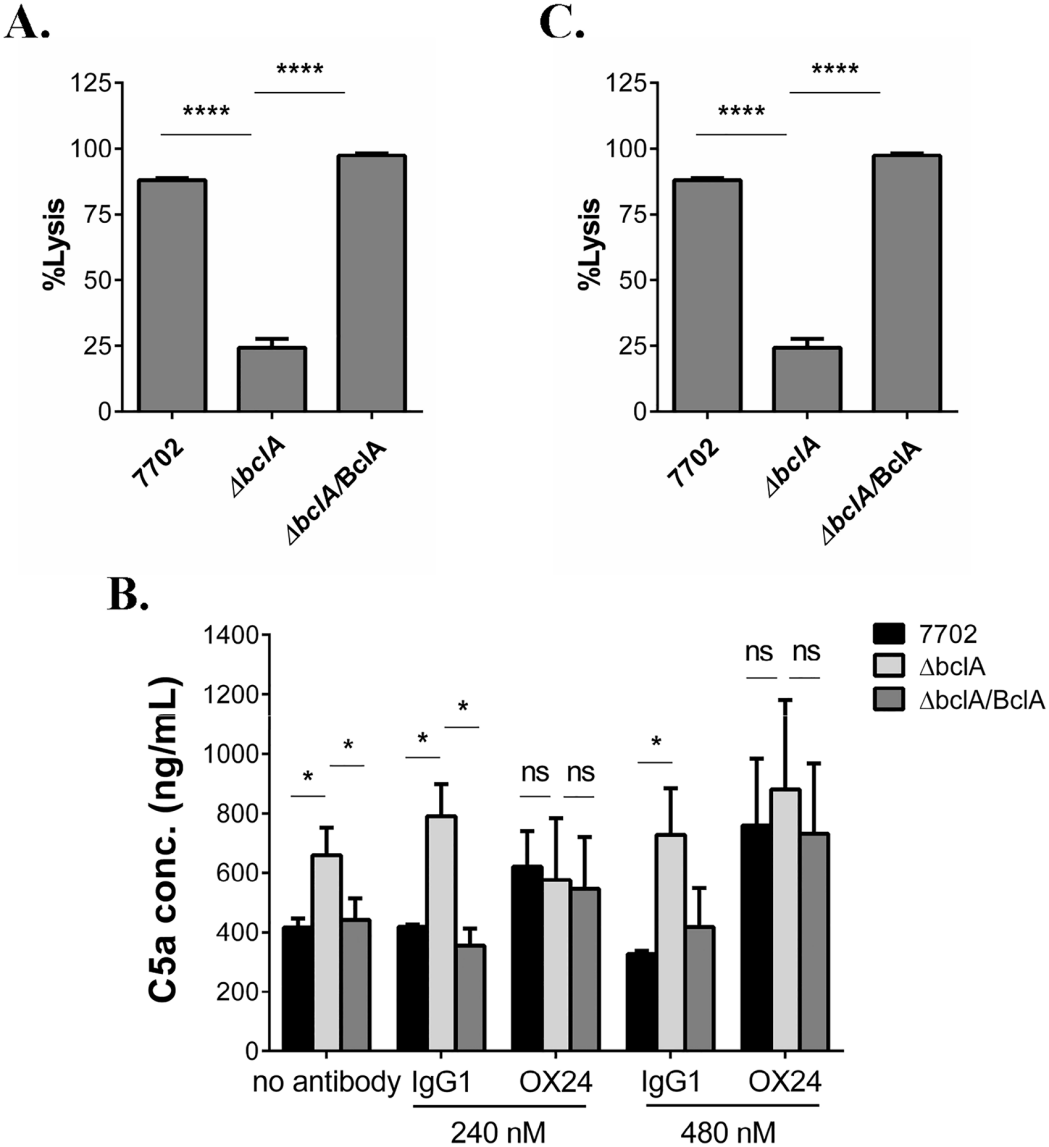
BclA-mediated CFH recruitment inhibited downstream complement activation *in vitro* and *in vivo*. (**A**) Complement hemolytic assay. Spores were incubated with 20% NHS and centrifuged. The supernatants (1:10 diluted) were used to perform complement hemolytic assays using opsonized sheep erythrocytes (EA-SRBC). Data shown was from at least three independent experiments. (**B**) Determination of C5a levels in human serum incubated with the different spores. GVB^0^ buffer containing 20% NHS was pre-treated with buffer only (no antibody), OX24, or control IgG1, followed by incubation with 7702, Δ*bclA* or Δ*bclA*/BclA spores. C5a levels in the supernatants were measured using the Human Complement Component C5a DuoSet. Data shown was combined from two independent experiments, each with duplicate wells. (**C**) Determination of C5a levels in mouse BAL fluid. C57BL/6 were i.n. inoculated with 7702 (n = 8), Δ*bclA* (n = 8), Δ*bclA*/BclA (n = 8) spores or PBS (n = 6). BAL fluids were collected 6 hours later and C5a level in the supernatant determined using the Mouse Complement Component C5a DuoSet. Data shown were combined from two independent experiments, each with duplicate wells. *, *p* < 0.05; **, *p* < 0.01. ****, *p* < 0.0001, *t* test.

Cleavage of C3b to iC3b prevents the formation of C5 convertase complexes that cleave C5 to C5a and C5b and the downstream formation of the membrane attack complex. Therefore, we next investigated the effect of BclA-mediated CFH recruitment on downstream complement activation. We first performed an indirect complement hemolytic activity assay to measure terminal stage complement activation [37]. NHS was preincubated with 7702, Δ*bclA* or Δ*bclA*/BclA spores and centrifuged. The supernatants were used as the source of complement for hemolysis assays using opsonized sheep erythrocytes (EA-SRBC) as the target. If BclA led to inhibition of downstream complement activation, 7702 or Δ*bclA*/BclA pre-incubated serum should contain more intact complement components than Δ*bclA* pre-incubated serum, and thus cause more hemolysis. We observed ~ 100% hemolytic killing of EA-SRBC in sera pre-incubated with 7702 and Δ*bclA*/BclA spores respectively, but only 20% in serum pre-incubated with Δ*bclA* spores (*p* < 0.0001) (Fig 3A).

We further measured the level of C5a in serum incubated with the different spores as a direct method to evaluate downstream complement activation. The results showed that the level of C5a was significantly higher in samples incubated with Δ*bclA* spores compared to those incubated with 7702 or Δ*bclA*/BclA spores (Fig 3B, no antibody). To determine if the inhibition was due to CFH, C5a assays were performed using OX24 or control antibody pre-treated serum. The results showed that pre-treatment with OX24 increased the C5a concentration in samples incubated with 7702 and Δ*bclA*/BclA spores, respectively, to a similar level as that seen in those incubated with Δ*bclA* spores (Fig 3B). In contrast, mouse IgG1 had no effect on the level of C5a in any of the samples. These results indicated that CFH was responsible for the apparent effect of BclA on C5a.

We next investigated the effect of BclA on C5a level *in vivo*. Mice were intranasally (i.n.) inoculated with the different spores. BAL fluids were then collected by lavaging the lungs with sterile PBS containing EDTA, which stops complement activation. We observed that C5a concentration in the BAL fluids from mice infected with 7702 or Δ*bclA*/BclA spores was significantly lower than that from mice infected with Δ*bclA* spores (Fig 3C). Taken together, the results described above indicated that BclA-CFH interaction led to reduced C5 cleavage both *in vitro* and *in vivo*.

### BclA significantly promoted spore persistence in the mouse lungs

**Fig 4:**
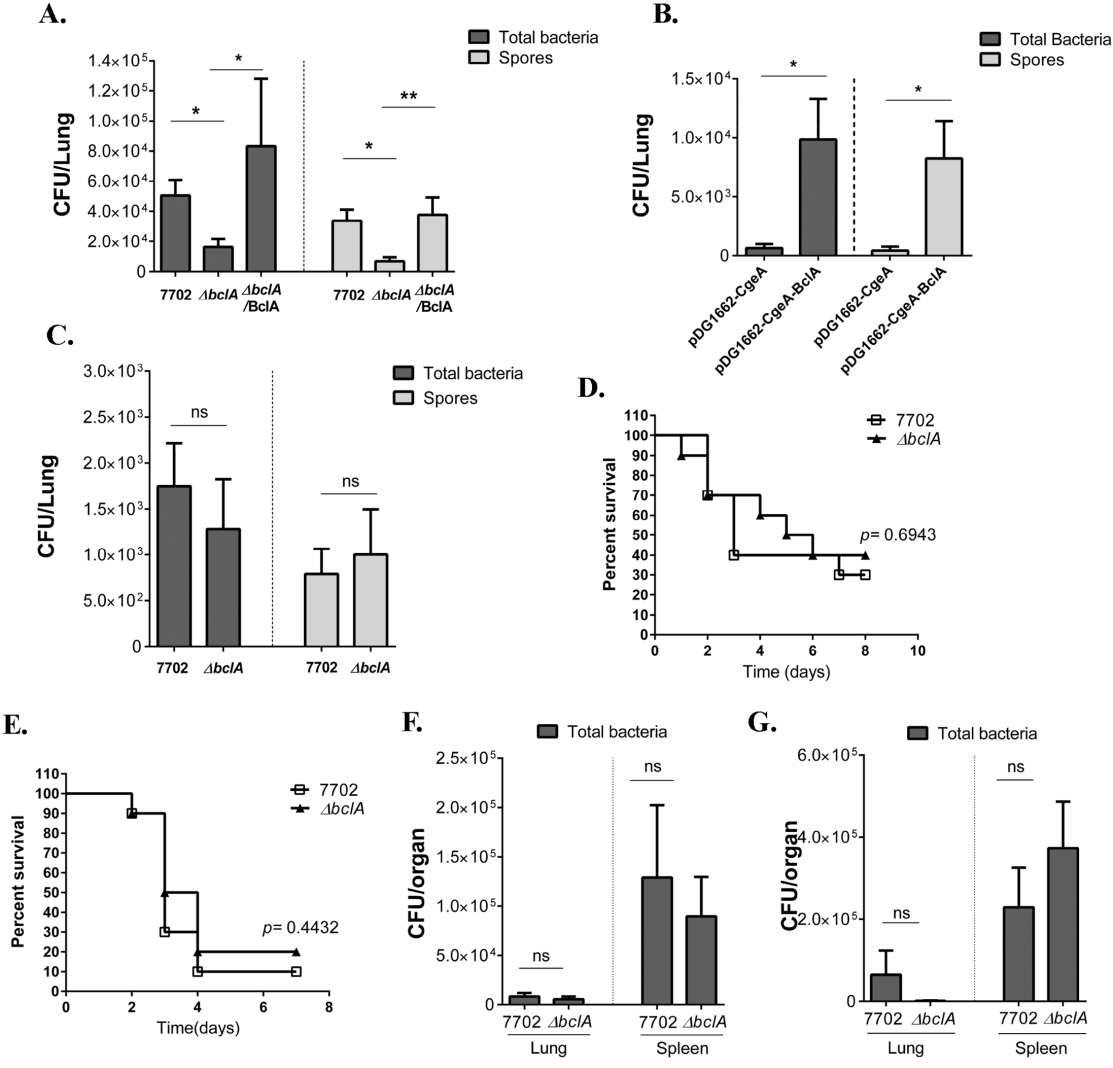
BclA significantly promoted spore persistence in the mouse lungs. Mice were i.n. inoculated with sub-lethal doses of various spores. Lungs were collected, homogenized and either dilution plated to determine the total viable bacterial counts, or heated at 68°C and dilution plated to determine the spore counts. (**A**). C57BL/6 mice were i.n. inoculated with ~1×10^8^ spores of 7702, Δ*bclA* or Δ*bclA*/BclA per mouse. Lungs were collected 2 weeks post inoculation. Data shown were combined from at least two independent experiments (7702, n = 12; Δ*bclA*, n = 7; Δ*bclA*/BclA, n = 4). (**B**) Balb/c mice were i.n. inoculated with ~ 1.5 ×10^7^ spores per mouse of *B*. *subitilis* containing pDG1662 vector (n = 20) or pDG1662-BclA (n = 20). Lungs were harvested at one week post inoculation. Data shown were combined from two independent experiments. (**C**). C3^-/-^ mice were more susceptible to *B*. *anthracis* than C57BL/6. Therefore, a sub-lethal dose of ~ 5×10^5^ spores/mouse was used for i.n. inoculation of C3^-/-^ mice. Lungs were collected at 2 weeks post inoculation. Data shown were combined from at least two independent experiments (7702, n = 14; Δ*bclA*, n = 12). (**D—G**) Mice were i.p. inoculated with lethal doses of 7702 or Δ*bclA* spores. (**D**) C57BL/6 mice were inoculated with ~1×10^8^ spores/mouse of 7702 (n = 10) or Δ*bclA* (n = 10) and survival monitored. Data shown were combined from two independent experiments. (**E**) C3^-/-^ mice were inoculated with ~5×10^6^ spores/mouse of 7702 (n = 11) or Δ*bclA* (n = 11). Data shown were combined from two independent experiments. (**F**) Bacterial burden in the lungs and spleen of C57BL/6 mice inoculated with ~1×10^8^ spores of 7702 (n = 10) or Δ*bclA* (n = 10) at 48 hours post inoculation. Data shown were combined from two independent experiments. (**G**) Bacterial burden in the lungs and spleen of C3^-/-^ mice inoculated with ~5×10^6^ spores of 7702 (n = 6) or Δ*bclA* (n = 4) at 48 hours post inoculation. Data shown were combined from two independent experiments. *, *p* < 0.05; **, *p* < 0.01; ****, *p* < 0.0001; *t* test. Analysis of survival curves was done using Log-rank test.

We first investigated if BclA was important for spore persistence. C57BL/6 mice were i.n. inoculated with sub-lethal doses of spores. Total bacteria and spore load in the lungs at two and four weeks post inoculation was determined. At both time points, C57BL/6 mice inoculated with 7702 spores harbored significantly more total bacteria and spores in the lungs than those inoculated with Δ*bclA* spores (Fig 4A and S5A Fig). Complementation of BclA in the Δ*bclA* background significantly increased the spore counts in the lungs at both time points. We tested the germination efficiency of 7702, Δ*bclA* and Δ*bclA*/BclA spores in three different media: a chemically defined germination media, LB and 100% NHS (S3 Fig). We did not observe any difference in the germination efficiency between the spores in any of the media. We also tested bacterial dissemination to distal organs such as the spleen and found no significant difference in bacterial burden in the spleen of mice inoculated with the different spores (S4A Fig). Hematoxylin and Eosin (H&E) staining was performed on lung sections from C57BL/6 mice collected at two weeks post i.n. inoculation with either 7702 or Δ*bclA* spores. Minimum pathology was observed in sections from both groups (S7 Fig), consistent with a previous report [4]. The alveolar and small airway epithelium appeared intact in both groups and lymphocyte infiltration was only occasionally observed. Overall, we did not see obvious differences in inflammatory responses in the lungs between the two groups.

To determine if BclA alone was sufficient to promote spore persistence in the lungs, we further examined *B*. *subtilis* spores expressing BclA. The results showed that expression of BclA on the surface of *B*. *subtilis* spores significantly increased both total bacteria and spore burden in the lungs compared to the vector control (Fig 4B). Together, these results suggested that BclA significantly promoted spore persistence *in vivo*.

### BclA promoted spore persistence in a complement-dependent manner

We next investigated the role of complement in spore persistence. CFH deficiency in mice caused uncontrolled complement activation resulting in C3 consumption [38]. Therefore, we compared spore persistence in C3^-/-^ mice, as C3 is where the three complement pathways converge. We reasoned that if BclA-mediated inhibition of complement was responsible for the increased spore persistence in the lungs, we should see no difference in spore persistence between 7702 and Δ*bclA* in C3^-/-^ mice. Indeed, no significant difference in either total bacteria or spore burden was observed in C3^-/-^ mice between 7702 and Δ*bclA*-infected groups at 2 or 4 weeks post inoculation (Fig 4C and S5B Fig), suggesting that BclA-mediated promotion of spore persistence was C3-dependent. It was previously reported that BclA bound complement component C1q. The binding leads to internalization of spores by epithelial cells through integrin α_2_β_1_ and opsonophagocytosis of spores by macrophages [30, 32]. We examined spore persistence in C1q-deficient (C1q^-/-^) mice. The results from C1q^-/-^ mice mirrored those from wild type C57BL/6 mice (S6A Fig), suggesting that BclA-C1q interaction was not important for spore persistence in the mouse lungs. Taken together, the results suggested that spore persistence was promoted by BclA-mediated inhibition of complement activation.

### BclA did not affect mouse survival or bacterial load in an acute lethal challenge model

To test if BclA contributes to virulence in acute infections, C57BL/6 and C3^-/-^ mice were injected with lethal doses of 7702 or Δ*bclA* spores by intraperitoneal injection (i.p.). No significant difference in mouse survival was observed between 7702 and Δ*bclA*-infected groups in either mouse strains (Fig 4D and 4E). We next compared the total bacteria and spore burden in the lungs and spleen of C57BL/6 and C3^-/-^ mice 48 hours post inoculation. We did not observe any significant difference in total bacteria or spore load in the lungs or the spleen between mice challenged with 7702 and those with Δ*bclA* spores (Fig 4F and 4G). These results indicate that BclA does not contribute to virulence in this lethal challenge model. This is consistent with results from previous studies using lethal infection models [29, 34]

### BclA inhibited antibody responses against *B*. *anthracis* spores in a complement-dependent manner

**Fig 5:**
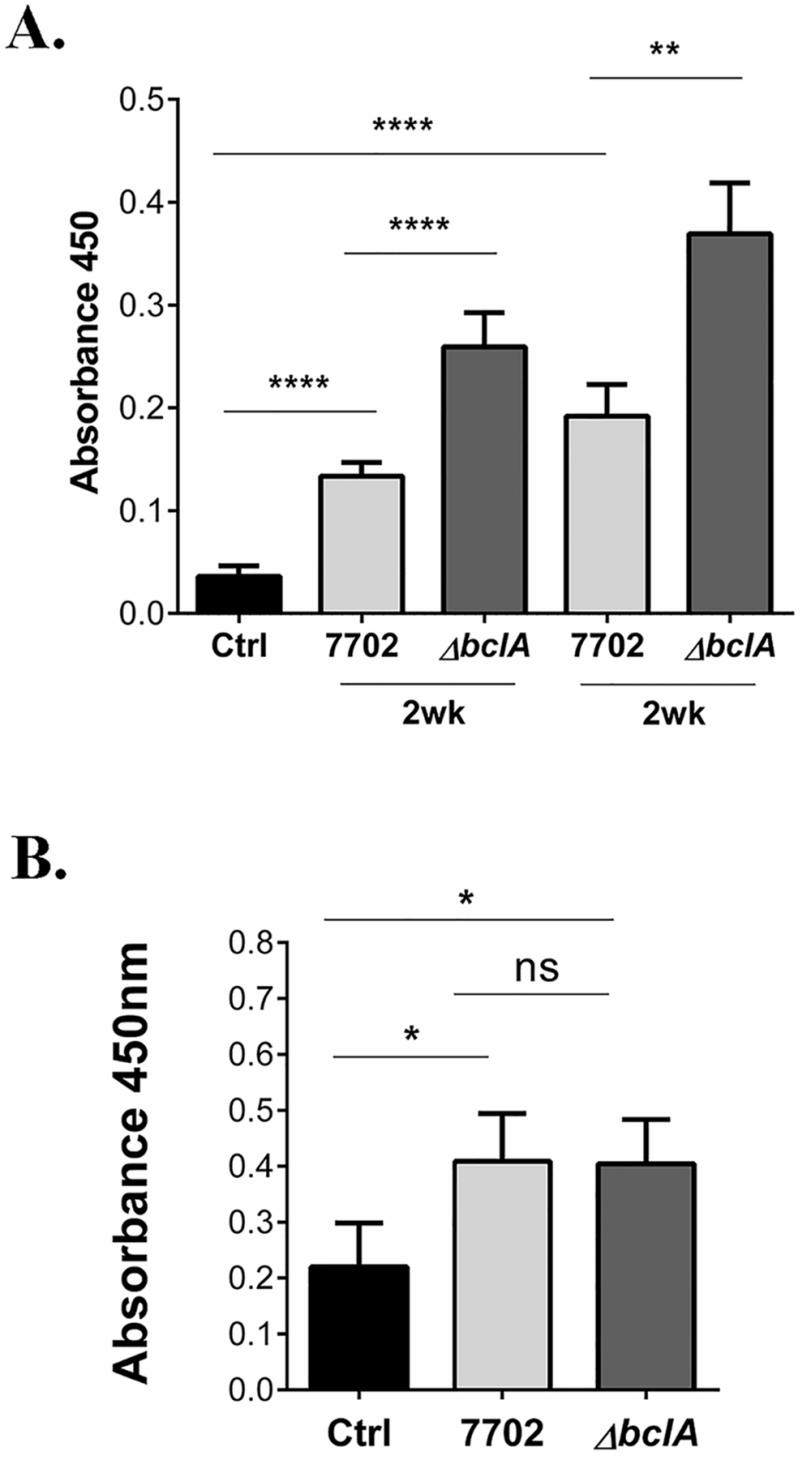
BclA inhibited antibody responses against spores. (**A**) C57BL/6 mice were i.n. inoculated with ~1×10^8^ spores of 7702, Δ*bclA* or vehicle control once and blood collected at 2 weeks post inoculation (2wk), or inoculated again with the same spores and dose at 2 weeks and blood collected at 4 weeks after the initial inoculation (4wk). Anti-spore antibodies in the serum were detected using ELISA. Data shown were combined from at least three independent experiments. The mouse number for the various groups is as follows: control, n = 8; 2wk experiment, n = 30 and 29 for 7702 and Δ*bclA*, respectively; 4wk experiment, n = 30 and 28 for for 7702 and Δ*bclA*, respectively. (**B**) C3^-/-^ mice were i.n. inoculated with vehicle control, or ~5×10^5^ spores of 7702 or Δ*bclA* and blood collected at 2 weeks post inoculation. Anti-spore antibodies in the serum were detected using ELISA. Data shown were combined from at least three independent experiments, with n = 10, 24 and 21 for control, 7702 and Δ*bclA*, respectively. *, *p* < 0.05; **, *p <* 0.01; ****, *p* < 0.0001; *t* test.

The complement system not only shapes the innate immune responses, but also guides the adaptive immune responses [39–43]. We examined the effect of BclA on host antibody responses against spores in the persistence model. Anti-spore IgG antibodies in serum from infected mice were detected using ELISA. Both 7702 and Δ*bclA* spores elicited specific antibody responses in C57BL/6 (Fig 5A), C3^-/-^ (Fig 5B) and C1q^-/-^ (S6B Fig) mice, respectively, compared to the saline control. However, C57BL/6 and C1q^-/-^ mice exposed to 7702 spores had significantly lower antibody titers compared to those exposed to Δ*bclA* spores (Fig 5A and S6B Fig), suggesting that BclA dampened antibody responses and that BclA-C1q interaction was not important in this process. The difference in anti-spore IgG titers between the 7702- and Δ*bclA*-infected groups was not detectable in C3^-/-^ mice (Fig 5B), suggesting that BclA dampened antibody responses against spores through downregulating C3 activation.

### BclA impaired protective immunity against lethal *B*. *anthracis* challenges

**Fig 6:**
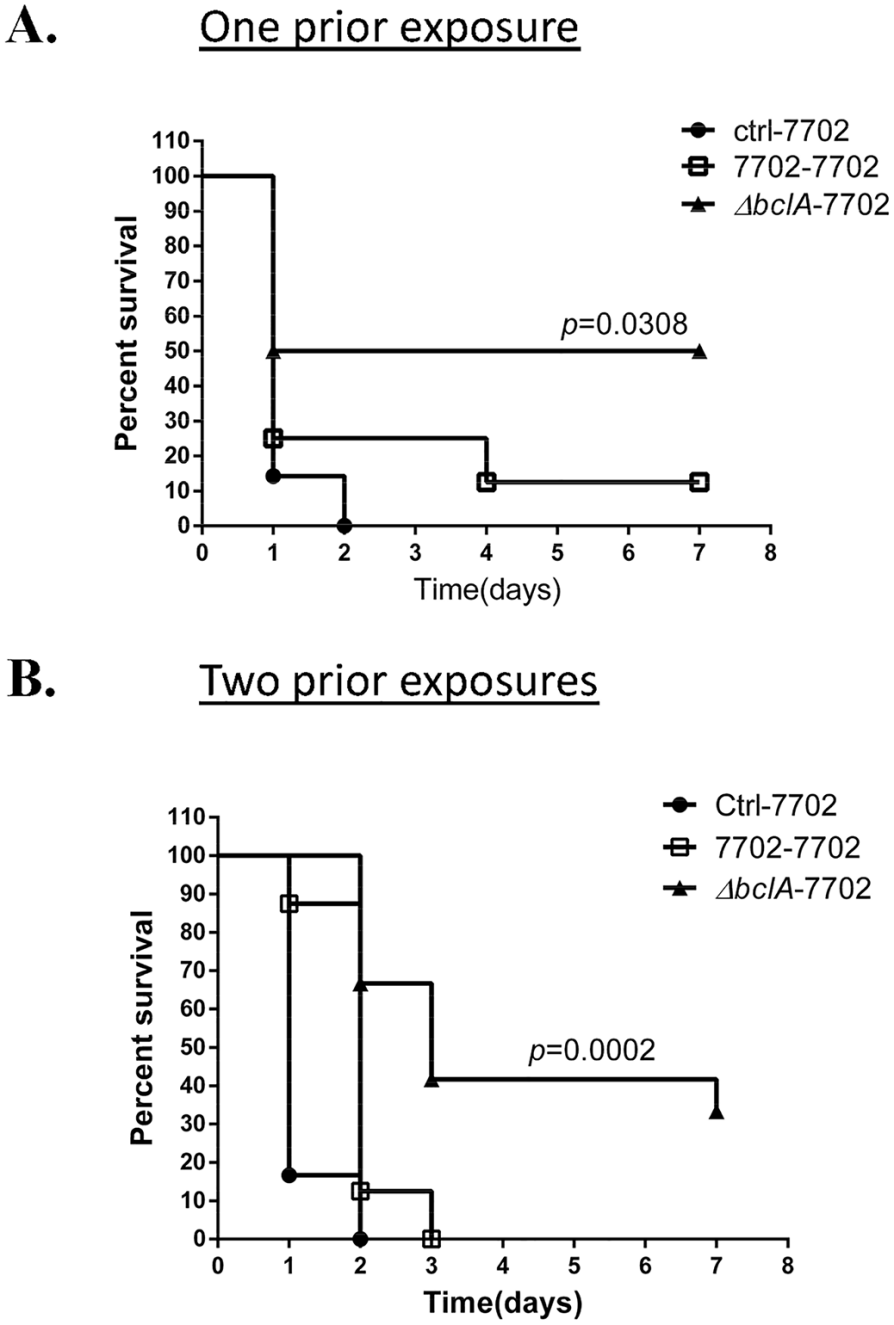
BclA impaired protective immunity against lethal *B*. *anthracis* challenges. C57BL/6 mice were i.n. inoculated with a sub-lethal dose of ~1×10^8^ spores of 7702, Δ*bclA* or vehicle control once (**A**) or twice (**B**). Mice were then challenged with ~1×10^10^ 7702 spores by i.p. injection 15 days after the last i.n. inoculation and monitored for survival. Data shown in (**A**) were combined from two independent experiments, with n = 7, 8, and 10 for ctrl-7702, 7702–7702, and Δ*bclA*-7702, respectively. Data shown in (**B**) were combined from two independent experiments, with n = 6, 8 and 12 for ctrl-7702, 7702–7702, and Δ*bclA*-7702, respectively. Log-rank test was used to statistical comparison of the survival curves.

Because of the significant difference in anti-spore antibody levels, we investigated if prior exposure to 7702 or Δ*bclA* spores triggered different protection against lethal *B*. *anthracis* challenges. In one set of experiment, C57BL/6 mice were i.n. inoculated with a sub-lethal dose of 7702 or Δ*bclA* spores and then challenged with a lethal dose of 7702 spores by intraperitoneal (i.p.) injection two weeks later. In another set, C57BL/6 mice were i.n. inoculated with sub-lethal doses of 7702 or Δ*bclA* spores at 0 and 2 weeks and then challenged with a lethal dose of 7702 spores by i.p. injection at four weeks. The results showed that for mice with one prior exposure (Fig 6A), those pre-exposed to 7702 spores succumbed to lethal challenges within two days, similar to those pre-exposed to saline only, whereas those pre-exposed to Δ*bclA* spores had a significantly better survival rate (*p* = 0.0308 vs. the saline control) with a median survival time of 4 days. For mice with two prior exposures (Fig 6B), the difference was even more pronounced (*p* = 0.0002 vs. the control group, *p* = 0.0069 vs. 7702 pre-exposed group). Taken together, these results suggested that BclA impaired protective immunity against lethal *B*. *anthracis* infections.

## Discussion

In this study we discovered a novel function for the major *B*. *anthracis* spore surface protein BclA. We demonstrated that BclA mediated recruitment of CFH to spores, facilitated C3b degradation on the spore surface, inhibited further C3 activation, and reduced C5 cleavage both *in vitro* and *in vivo*. We further showed that BclA promoted spore persistence in the host lungs and inhibited antibody responses against spores in a C3-dependent manner. Furthermore, BclA impaired protective immunity against lethal *B*. *anthracis* challenges. These results describe for the first time a spore-mediated immune modulatory mechanism through inhibition of complement. The results also suggested an important role of complement in persistent infections, an aspect of pathogen-complement interaction that is poorly understood.

The ability of BclA to mediate CFH binding was demonstrated by 1) Δ*bclA* spores bound significantly less CFH than the parent spores, and the defect was restored by complementing BclA, 2) BclA expressed on the surface of *B*. *subtilis* spores was sufficient to promote CFH binding, and 3) recombinant BclA protein bound to purified CFH in a concentration-dependent manner. We observed weaker CFH binding by Δ*bclA* and *B*. *subtilis* control spores. It is possible that there is another unknown low-affinity CFH binding protein on these spores or non-specific binding of CFH to spores. Our results also suggest that recognition of CFH by BclA is not human specific, *i*.*e*., BclA can bind both human and murine CFH, unlike some other CFH binding proteins such as the CFH-binding protein (fHbp) of *Neisseria meningitidis* [44] and PspC of *Streptococcus pneumoniae* [45]. A group A streptococcal collagen-like protein (Scl1) was reported to bind CFH via the C-terminal variable region of Scl1 [46]. While BclA is also a collagen-like protein, sequence comparison indicated no significant sequence similarities between the two proteins beyond the GXY triplet-repeating motif. BclA also did not show any significant sequence similarities to other reported microbial CFH binding proteins. Thus BclA is a novel CFH binding protein. BclA-bound CFH retained its co-factor activity, as shown by increased C3b degradation on the surface of parent and complemented spores compared with Δ*bclA* spores. BclA-CFH interaction inhibited further C3 activation, and decreased C5 activation as shown by C5a ELISA and hemolytic assays. The finding that C5 cleavage was also reduced in the mouse lungs in the presence of BclA further suggested that this effect was relevant *in vivo*. In addition, CFH functional blocking antibodies completely abolished the complement inhibitory activity of BclA, suggesting that the BclA-CFH interaction was responsible for this activity.

It has been known for decades that *B*. *anthracis* spores were able to persist in the host lungs for prolonged periods of time. This capability was thought to be due to the dormancy and resilience of spores. The results from this study describe for the first time a specific persistence-promoting mechanism mediated by the spore surface protein BclA. The observation that the difference in spore load in the lungs between 7702 and Δ*bclA*-infected mice disappeared in C3^-/-^ mice suggests BclA promotes spore persistence in the lungs by inhibiting complement activities. It was previously reported that BclA directly binds C1q and this interaction leads to activation of the classical complement pathway and opsonophagocytosis of spores by macrophages [30, 32]. However, the results obtained from C1q^-/-^ mice suggest that BclA-C1q interaction is not important for spore persistence or antibody response to spores. This suggests that in this model system, inhibition of the alternative pathway plays a dominant role in promoting spore persistence. Recently it was shown that binding of CFH to the PspC protein of *S*. *pneumoniae* promoted pneumococcal nasal colonization by CFH-mediated bacterial adherence to the epithelium [47]. In our case, CFH is present in C3^-/-^ mice, suggesting that BclA-mediated promotion of spore persistence ultimately depends on C3 and works by inhibiting complement activities. Thus the persistence colonization mechanism described here is distinct from that of *S*. *pneumoniae*. The percentage of bacteria recovered from the lungs at 2 weeks post inoculation versus the initial inoculum was ~ 0.05%. This is in the same range as reported previously in Balb/c mice (~ 0.08%) [4].

The role of BclA in pathogenesis has been controversial despite the fact that it is a dominant protein on the spore surface. Studies in lethal infection models did not show any contribution of BclA to virulence [29, 34]. In the lethal spore challenge model here, our results also show no difference in either mouse survival or bacterial burden between mice challenged with 7702 and Δ*bclA* spores, consistent with previous studies. The findings here suggest that the primary role of BclA *in vivo* may be to promote the long term survival of spores through inhibition of complement activities.

We observed that 7702 spores led to significantly lower anti-spore antibody levels compared to Δ*bclA* spores in C57BL/6 and C1q^-/-^ mice. The reduced antibody response was not due to a lower bacterial burden of 7702 *in vivo*; on the contrary 7702 infected mice had a higher spore burden in the lungs and a similar burden in the spleen compared to Δ*bclA*-infected mice. The fact that there was no difference in antibody responses in C3^-/-^ mice suggested that BclA-mediated inhibition of C3 and/or downstream complement activation was responsible for the reduced antibody response to spores. The complement system influences B cells, T cells and antigen-presenting cells, the major cell types in the adaptive immune system [48–57]. The interaction between BclA and CFH can potentially affect all these components of the adaptive immune system. It has also been reported that CFH binding led to impaired antibody responses against the corresponding CFH-binding protein [47, 58–61]. Antibodies tend to recognize epitopes outside the CFH binding sites hence do not block CFH binding, or even enhance CFH binding. It would be interesting to investigate how BclA-CFH interaction affects antibody responses against *B*. *anthracis* spores.

Finally we observed that pre-exposure to 7702 spores conferred virtually no protection against lethal challenges whereas pre-exposure to Δ*bclA* spores provided significant protection in our infection model. The finding that BclA not only inhibited antibody responses against spores but also impaired protective immunity against *B*. *anthracis* lethal challenges has important implications in anthrax vaccine development and in persistent infections in general. With respect to vaccine development, BclA has been pursued as a vaccine candidate together with protective antigen (PA) as a multicomponent anthrax vaccine. Vaccination with BclA either as a recombinant protein or as a DNA vaccine augmented the protective efficacy of PA [62–64]. However, vaccination with formalin killed spores showed that Δ*bclA* spores provided greater protection than BclA-producing spores [65]. Our findings here suggest that the latter observation may be due to the effect of BclA on complement. The fHbp of *N*. *meningitidis* was approved as a component in multicomponent vaccines against serogroup B meningococcus [66–68]. Recent studies found that fHbp mutant proteins defective in CFH binding were more immunogenic and elicited stronger protective antibody responses than wild type proteins [59, 60, 69]. This raised the possibility that perhaps BclA mutants defective in CFH binding may offer better protection against anthrax infections.

Previous studies on the effect of pathogen manipulation of complement have been primarily focused on the more immediate effects of complement such as complement-mediated killing and opsonophagocytosis in the context of acute infections. For those bacteria that are susceptible to complement-mediated killing such as Gram-negative pathogens (*e*.*g*., *N*. *meningitidis*) or spirochetes (*e*.*g*., *Borrelia burgforderi*), inhibition of complement activation by recruiting CFH or other mechanisms confers serum resistance to the bacteria and is important for bacterial survival and virulence *in vivo* [66, 70–72]. For Gram-positive bacteria which are relatively resistant to serum killing due to their thick peptidoglycan cell wall, inhibition of complement activation can hinder phagocytosis and protects bacteria from phagocytic clearance. For example, *Streptococcus pyogenes* was found to inhibit phagocytosis by inactivating C3b in a strain-dependent mechanism [73]. Binding to CFH or C4-binding protein by *S*. *pyogenes* led to increased mortality in mouse models [74]. In contrast, the long-term effect of complement inhibition by pathogens has not been well studied in a systematic manner. The results presented here suggest that inhibition of complement by pathogens can play an important role in promoting persistent infections. In addition, because spores are resistant to lysis by complement and to phagocytic killing [30, 75], the role of complement observed in this study was likely due to the indirect activities of the complement effectors on the innate and adaptive immune system. This finding is particularly relevant to Gram-positive, encapsulated, or spore-forming pathogens, which tend to be relatively resistant to complement-mediated or phagocytic killing. With respect to how inhibition of complement promotes spore persistence, there may be multiple mechanisms involving both the innate and adaptive immune systems. Decreased production of C3a and C5a can affect cytokine production and the activation status of phagocytes. CFH binding to spores may not only dampen antibody responses but also affect the specific antibodies produced, as found in *N*. *meningitidis*. In addition, T cell and/or B cell functions can be affected [48–57]. Further studies to elucidate the detailed mechanism underlying the role of complement in persistent infections will be important.

In conclusion, we characterized the first CFH-binding protein of *B*. *anthracis* and described for the first time a spore-mediated immune inhibition mechanism of *B*. *anthracis*. These results shed light on the role of BclA *in vivo*. In addition, our findings suggest that in addition to conferring resistance to complement-mediated killing and opsonophagocytosis, complement inhibition by pathogens have long-term consequences with respect to persistent infections and protective immunity. Considering a growing list of microbial pathogens capable of modulating complement activities [76–80], our findings have broad implications.

## Methods and Materials

### Bacterial strains, spores, and reagents

Strains and plasmids used in this study are listed in S1 Table. Spores of *B*. *anthracis* and *B*. *subtilis* were prepared by culturing in a PA broth or on LB agar plates as described [4, 30]. To inhibit spore germination, a germination inhibitor D-alanine (2.5 mM) was included in solutions for assays involving spores. Normal human serum (NHS), GVB^0^ buffer (Gelatin Veronal Buffer without Ca^2+^, Mg^2+^, 0.1% gelatin, 5 mM Veronal, 145 mM NaCl, 0.025% NaN_3_, pH 7.3), VBS^++^ buffer (5 mM Veronal, 145 mM NaCl, 0.025% NaN_3_, pH 7.3, 0.15 mM CaCl_2_ and 0.5 mM MgCl_2_), purified complement proteins and goat anti-human CFH, anti-human C1q and anti-human C3 antibodies, were from Complement Technology unless otherwise stated. Secondary antibodies were from Thermo Fisher Scientific unless otherwise stated. 2,2,2-Tribromoethanol (Avertin), bovine serum albumin (BSA), chicken ovalbumin (OVA), D-alanine, and L-alanine were purchased from Sigma. Heat inactivation of complement was carried out at 56°C for 30 min.

### Complementation of BclA mutant strain and heterologous expression of BclA on the surface of *B*. *subtilis* spores

A DNA fragment containing the *bclA* gene and its upstream sequence (~ 1kb) was cloned into an *E*. *coli—B*. *anthracis* shuttle vector pUTE583 [81]. The construct was then introduced into Δ*bclA* by electroporation as described previously [82]. To express BclA on the surface of *B*. *subtilis* spores, a DNA fragment encoding amino acid residues 39–400 of BclA was fused to the C-terminus of CgeA, a protein on the outermost surface of *B*. *subtilis* spores [83]. The first 38 amino acid residues were omitted because this region was reported to be proteolytically cleaved before anchorage of BclA onto the *B*. *anthracis* spore surface [84]. CgeA-BclA fusion was cloned into pDG1662, which allows the ectopic integration at the non-essential *amyE* locus in the *B*. *subtilis* chromosome [85, 86]. Surface expression was evaluated by staining spores with anti-BclA antibodies and fluorescently labeled secondary antibodies followed by immunofluorescence microscopy or flow cytometry analysis, as described in S1 Text.

### Spore pull down assays

To detect CFH recruitment to the spore surface, **~** 5×10^7^ spores were incubated at 37°C for 30 min in PBS containing 2.5 mM D-alanine and supplemented with one of the following: purified human CFH (10 μg/ml), bovine serum albumin (10 μg/ml), 10% (v/v) heat-inactivated NHS, 10% (v/v) heat-inactivated mouse serum (from C57BL/6), or heat-inactivated mouse BAL fluid (from C57BL/6). The spores were then washed three times with ice-cold PBS containing 2.5 mM D-alanine and resuspended in the same buffer. An aliquot of the spore suspension was used to titer the spores by dilution plating and the rest were frozen until ready for analysis. Equal amounts of spores were boiled in SDS-sample loading buffer and the supernatants were subjected to Western blot analysis using goat anti-human CFH (1:10000) or sheep anti-CFH antibody (1:2000, Abcam) followed by incubation with rabbit anti-goat antibody conjugated to horseradish peroxidase (HRP) (1:10000, Invitrogen) or HRP-conjugated rabbit anti-sheep IgG (1:10000, Invitrogen) for 1 hr.

To detect iC3b deposition, **~** 5×10^7^ spores were incubated in PBS buffer containing 500 μg/ml C3b, 100 μg/ml CFH, 4 μg/ml CFI, 0.1% BSA, 1 mM MgCl_2_ and 2.5 mM D-alanine at 37°C for 10 min. Spores were washed three times with ice-cold PBS containing 2.5 mM D-alanine. Equal amounts of spores were subject to Western blot analysis following the procedure described above. C3 fragments were detected using goat-anti human C3 (1:10000) and rabbit anti-goat HRP (1:10000). Band intensities were quantified using Image J.

### Flow cytometry to determine CFH binding and iC3b deposition

For CFH binding, **~** 5×10^7^ spores were incubated in buffer containing 2.5 mM D-alanine and supplemented with either 25 μg/ml purified human CFH or 10% heat inactivated NHS at 37°C for indicated length of time. Spores were then washed and fixed with 2% paraformaldehyde for 20 min at room temperature. Bound CFH was detected using goat anti-human CFH (1:400, Santa Cruz) followed by donkey anti-goat PE (1:400, Santa Cruz). For iC3b deposition, spores were incubated in buffer containing 10% NHS and 2.5 mM D-alanine for indicated length of time. iC3b was detected using mouse monoclonal antibody to human iC3b (neoantigen) (1:400; Quidel) and donkey anti-mouse 647 (1:400; SantaCruz). Samples were analyzed in a two laser Accuri C6 analytical flow cytometer using forward and side scatter parameters to gate on at least 20,000 spores. The red laser was used to measure the mean fluorescence intensity (MFI) of PE-labeled samples and data were analyzed using CFlow Plus (Accuri Cytometers) and graphed using GraphPad Prism 6 analysis software.

### Solid phase binding assays

Recombinant BclA (rBclA) was purified as described previously [32]. Microtiter 2HB plates were coated with 10 ug/ml purified CFH or ovalbumin (OVA) in HBS (20 mM HEPES and 50 mM NaCl, pH 7.4) overnight at 4°C. The wells were washed to remove unbound proteins by HBS with 0.05% Tween 20 (HBST), blocked in HBST with 1% OVA for 1 hr at room temperature, and incubated with increased concentrations (0.01, 0.1, 1, 5, 15 and 30 μM) of His-tagged rBclA in HBST for 2 hrs at room temperature. The wells were then washed three times with HBST and incubated with anti-His HRP (1:3000, Alpha Diagnostic Intl. Inc.) for 1 hr. Plates were developed with Sigmafast OPD and read at 450 nm. Apparent *K*_D_ was determined by non-linear regression (GraphPad Prism 6).

For spore binding, purified human CFH was immobilized onto wells of 96-well plates, blocked and incubated with ~ 1×10^7^ biotin-labeled spores suspended in the blocking buffer supplemented with 2.5mM D-alanine for 30 min at 37°C followed by washing and incubation with streptavidin-conjugated to HRP.

### Determination of C3a and C5a levels *in vitro*

Approximately 5×10^7^–1×10^8^ spores were incubated in GVB^0^ buffer containing 20% NHS and 2.5 mM D-alanine at 37°C for 30–60 min. Complement activation was terminated by adding 50 mM EDTA. The samples were centrifuged to remove spores. C3a and C5a levels in the supernatants were determined using Human C3a ELISA kit (BD OptEIA^™^) and Human Complement Component C5a DuoSet (R&D), respectively. To determine the effect of CFH functional blocking antibody OX24, 20% NHS in GVB^0^ buffer was pre-incubated with OX24 (Pierce Antibody) or isotype control mouse IgG1 (Sigma) at 240 nM or 480 nM final concentration at 37°C for 30 min. The reaction mix was then incubated with spores as described above.

### Complement hemolytic assay

Spores were incubated in buffer containing 20% NHS and 2.5 mM D-alanine at 37°C for 60 min. After centrifugation, the supernatants were diluted (1:10) in VBS^++^ (Ca^2+^, Mg^2+^) and used as the source of complement in hemolytic assays with opsonized sheep erythrocytes (1×10^7^ cells) following the instructions of the supplier (EA-SRBC, CompTech). %Lysis is calculated as $\frac{{\text{OD}}_{540}\left(\text{test}\right)-{\text{OD}}_{540}\left(\text{Blank}\right)}{{\text{OD}}_{540}\left(\text{total lysis}\right)-{\text{OD}}_{540}\left(\text{Blank}\right)}\;\times 100$.

### Determination of C5a levels *in vivo*

C57BL/6 mice were i.n. inoculated with different spores (~1×10^8^ spores/mouse) and BAL fluid was collected 6 hours later by lavaging the lungs with 1ml cold sterile PBS containing 50 mM EDTA. The lavage fluids were centrifuged to remove cells and bacteria. C5a level in the supernatants was measured using the Mouse Complement Component C5a DuoSet (R&D).

### Mouse infection and determination of bacterial burden

All animal procedures were performed according to protocols approved by the Institutional Animal Care and Use Committee, Texas A&M Health Science Center (TAMHSC). C57BL/6 (originally purchased from the Jackson Laboratories), C1q^-/-^ [87] and C3^-/-^ [88] mice were maintained at the animal facility at TAMHSC. Mice were euthanized by i.p. injection with an overdose of 2,2,2-Tribromoethanol (Avertin) followed by terminal bleed. Intranasal inoculation was performed as previously described [4]. Briefly, 6–12 week old mice were anesthetized with Avertin (0.3 mg/g body weight) and then inoculated with 20 μls of an indicated sub-lethal dose of spores. For lethal challenge experiments, mice were inoculated with a lethal dose of spores by i.p. injection. Mice were monitored for survival and other symptoms daily. Both male and female mice were used in the experiments in a sex-matched manner.

Lungs and spleens were homogenized in 1 ml sterile ice-cold PBS containing 2.5 mM D-alanine, and either directly dilution plated to determine the total bacterial counts or heated at 68°C for 60 min and dilution plated to determine the spore counts. Lungs were also fixed for histological evaluation as described in S1 Text.

### Determine serum IgG levels by ELISA

Total exosporium proteins were extracted from 7702 spores as previously described [89] with slight modifications. Briefly, ~5×10^9^ spores were resuspended in 200 μl of an extraction buffer (50 mM Tris-HCl, pH 7.4, 8 M urea, and 2% (v/v) 2-mercaptoethanol), heated for 20 min at 90°C, and centrifuged at 13,000× g for 10 min. The supernatant was then treated with 20% (v/v) ice-cold trichloroacetic acid for 30 min on ice and centrifuged at 13,000×g at 4°C for 25 min. The pellet was washed once with 1 ml ice-cold acetone, centrifuged at 7000 r.p.m for 2 min, and dissolved in 100 μl of a solution of 200 mM Tris-HCl (pH 7.4) and 0.1 M glycine.

Blood was collected either from the saphenous vein, or by terminal bleed from the posterior vena cava, at two weeks after mice were inoculated with spores. Blood was allowed to clot at room temperature for 45 min before centrifugation at 4000 r.p.m. at 4°C for 10 min. Serum was either stored immediately at -80°C or at 4°C with 0.1% sodium azide. Extracted spore antigens were immobilized onto 96-well plates at 0.5 μg/well. The plates were washed twice with PBS containing 0.1% Tween-20 (PBST), and blocked with PBST containing 3% BSA at 37°C for 1 hr. Serum samples were diluted (1:100 for serum from C57BL/6 and C1q^-/-^, and 1:2 for serum from C3^-/-^ mice) with PBS containing 3% BSA and incubated at 37°C for 1 hr. The wells were washed three times with PBST. Bound IgG was detected using goat anti-mouse IgG conjugated with HRP (1:2500, Invitrogen).

### Spore germination

Spore germination was evaluated as described in S1 Text.

### Statistical analysis

Pairwise comparison was carried out using Student’s *t* test. Survival analysis was performed using the Log-rank test (GraphPad Prism 6).

### Ethics statement

All animal experiments were performed in accordance to procedures approved by the Institutional Animal Care and Use Committee at Texas A&M Health Science Center (IACUC# 2015-0361-IBT). The Texas A&M University Health Science Center—Institute of Biosciences and Technology is registered with the Office of Laboratory Animal Welfare per Assurance A4012-01. It is guided by the PHS Policy on Human Care and Use of Laboratory Animals (Policy), as well as all applicable provisions of the Animal Welfare Act. Mice were euthanized by intraperitoneal injection of overdosed Tribromethanol/Avertin followed by terminal bleed. Mice were anesthetized with Avertin before intranasal inoculation of spores. All efforts were made to minimize animal suffering.

## Supporting Information

S1 Text Supporting methods and materials. (DOCX)

S1 Table Strains and plasmids used in this study. (DOCX)

S1 Fig Expression of BclA on the surface of *B*. *anthracis* and *B*. *subtilis* spores. Immunofluorescence microscopy and flow cytometry was performed as described in Supporting Methods and Materials. **A**. Imunofluorescence microscopy of spores of *B*. *anthracis* 7702, Δ*bclA*, Δ*bclA*/BclA, and *B*. *subtilis* pDG1662-CgeA vector only and *B*. *subtilis* pDG1662-CgeA-BclA. Spores were labeled with Texas Red to visualize spores. They were then incubated with rabbit anti-BclA antiserum and goat anti-rabbit antibodies conjugated to Alexa Fluor 488. **B**. Flow cytometry analysis. Spores were incubated with anti-BclA antiserum and secondary antibodies conjugated to Alexa Fluor 594, or with secondary antibodies only. **C**. Spores were incubated with rabbit pre-bleed serum and secondary antibodies conjugated to Alexa Fluor 594. The spores were then examined by phase contrast and fluorescence microscopy.
(TIF)

S2 Fig Recruitment of CFH from heat-inactivated serum and BAL fluids. Spores of *B*. *anthracis* 7702 and Δ*bclA*, and *B*. *subtilis* (*B*. *sub*), *B*. *subtilis* carrying pDG1662-CgeA vector control (pDG1662) and pDG1662-CgeA-BclA (pDG1662-BclA) were incubated in 10% heat-inactivated mouse serum (**A** and **B**), 10% heat-inactivated human serum (**C** and **D**) or heat-inactivated BAL fluid (**E** and **F**) at 37°C for 30 min. Spore-bound CFH was detected using pull down assays as described in the Methods and Materials section. Different amounts of spores (~5×10^6^, 5×10^7^ and 5×10^8^ spores) were used for the experiment in panel A, and ~ 5×10^7^ spores were used for experiments in panels B—F. Data shown were from representative experiments.
(TIF)

S3 Fig Germination of 7702, Δ*bclA* and Δ*bclA*/BclA spores. Spores were heat activated at 68°C for 30 min and resuspended in a germination buffer (50 mM Tris-HCl, pH 7.4, 10 mM NaCl, 100 mM L-alanine) (**A**), LB (**B**) or NHS (**C**) to reach OD_580_ of 1.0. Kinetic readings were performed every 5 or 10 min at 37°C for 60 min using a Synergy H1 Multi-Mode Reader. The experiment was performed twice, each with duplicate wells. Data was normalized to OD at time zero.
(TIF)

S4 Fig Bacteria and spore burden in the mouse spleen at two weeks post inoculation. C57BL/6 (**A**) and C1q^-/-^ (**B**) mice were i.n. inoculated with ~1×10^8^ spores per mouse. C3^-/-^ (**C**) mice were i.n. inoculated with ~ 5×10^5^ spores per mouse. Spleens were collected at 2 weeks post inoculation and homogenized in 1ml sterile PBS containing 2.5 mM D-alanine. The homogenates were either plated directly to determine the total viable bacterial counts or heated at 68°C for 1 hr and dilution plated to determine spore counts. Data shown were combined from at least two independent experiments. C57BL/6, n = 12 and 7 for 7702 and Δ*bclA*, respectively; C1q^-/-^, n = 6 and 5 for 7702 and Δ*bclA*, respectively; C3^-/-^, n = 6 and 5 for 7702 and Δ*bclA*, respectively.
(TIF)

S5 Fig Bacteria and spore load in the mouse lungs at 4 weeks post inoculation. This was performed as described in the legend for Fig 4 except that lungs were collected at 4 weeks post inoculation. C57BL/6 (**A**) and C3^-/-^ (**B**) mice were i.n. inoculated with sub-lethal doses of spores of 7702, Δ*bclA* or Δ*bclA*/BclA. Bacterial and spore load in the lungs at 4 weeks post inoculation was determined. Data shown were combined from at least two independent experiments. C57BL/6 mice, n = 14, 15, and 14 for 7702, Δ*bclA* and Δ*bclA*/BclA, respectively; C3^-/-^ mice, n = 9 and 7 for 770 and Δ*bclA*, respectively. **, *p* < 0.01; ****, *p* < 0.0001; *t* test.
(TIF)

S6 Fig Bacteria and spore burden in the lungs, and serum antibody titers in C1q^-/-^ mice. (**A**) C1q^-/-^ mice were i.n. inoculated with ~ 1×10^8^ spores per mouse of 7702 (n = 7), Δ*bclA* (n = 9) or Δ*bclA*/BclA (n = 5). Lungs were collected at 2 weeks post inoculation, and total viable bacteria and spore counts determined. Data shown were combined from two independent experiments. (**B**) C1q^-/-^ mice were i.n. inoculated with ~ 1×10^8^ spores per mouse of 7702, Δ*bclA* or vehicle control once (2wk) or twice (4wk). Blood was collected 2 weeks after the last inoculation. Antibody titers in the serum were measured using ELISA with spore protein extracts as antigens. Data shown were combined from at least two independent experiments. Ctrl, n = 6; 7702-2wk, n = 17; Δ*bclA* (2wk), n = 12; 7702-4wk, n = 17; Δ*bclA* (2wk), n = 12. *, *p* < 0.05; **, *p <* 0.01; ***, *p* < 0.001; ****, *p* < 0.0001; *t* test.
(TIF)

S7 Fig H&E stained lung sections from C57BL/6 mice inoculated with spores. C57BL/6 mice were i.n. inoculated with 1×10^8^ 7702 or Δ*bclA* spores and lungs collected at 2 weeks post inoculation. Representative images of lung sections from 7702 (**A**) and Δ*bclA* (**B**)-inoculated mice (n = 2/group) are shown.
(TIF)
