# Correction: *Bacillus anthracis* Spore Surface Protein BclA Mediates Complement Factor H Binding to Spores and Promotes Spore Persistence

**DOI:** 10.1371/journal.ppat.1005968

**Published:** 2016-10-13

**Authors:** Yanyu Wang, Sarah A. Jenkins, Chunfang Gu, Ankita Shree, Margarita Martinez-Moczygemba, Jennifer Herold, Marina Botto, Rick A. Wetsel, Yi Xu

In Fig 3, the panel A graph was duplicated in panel C. Additionally, in Fig 5, panel A, the X axis was incorrectly labeled as “2wk” instead of “4wk”. Please see the corrected figures here.

**Fig 3:**
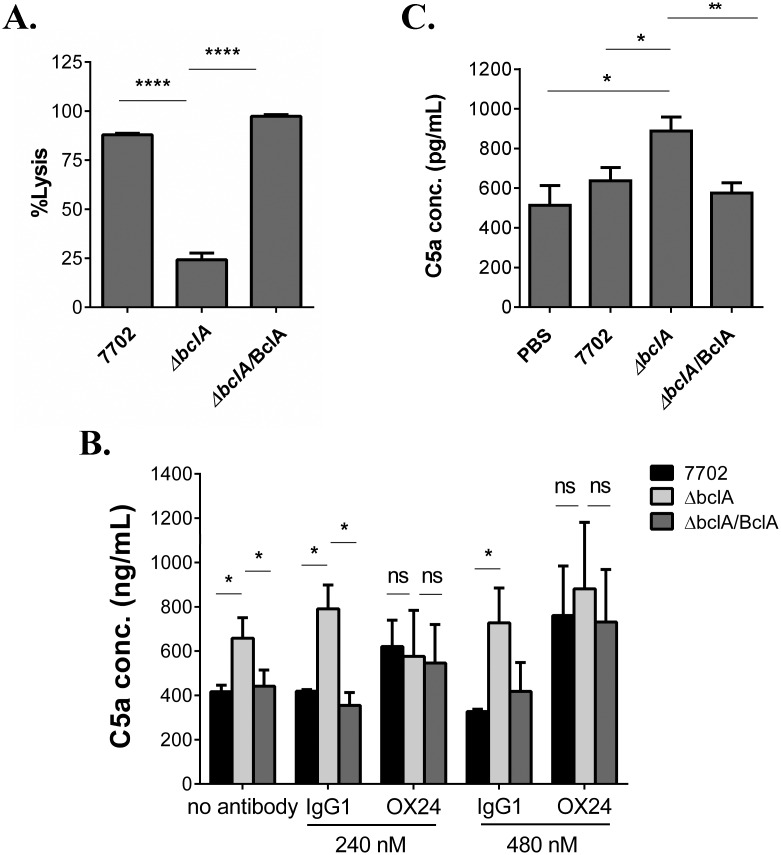
BclA-mediated CFH recruitment inhibited downstream complement activation in vitro and in vivo. (**A**) Complement hemolytic assay. Spores were incubated with 20% NHS and centrifuged. The supernatants (1:10 diluted) were used to perform complement hemolytic assays using opsonized sheep erythrocytes (EA-SRBC). Data shown was from at least three independent experiments. (**B**) Determination of C5a levels in human serum incubated with the different spores. GVB0 buffer containing 20% NHS was pre-treated with buffer only (no antibody), OX24, or control IgG1, followed by incubation with 7702, ΔbclA or ΔbclA/BclA spores. C5a levels in the supernatants were measured using the Human Complement Component C5a DuoSet. Data shown was combined from two independent experiments, each with duplicate wells. (C) Determination of C5a levels in mouse BAL fluid. C57BL/6 were i.n. inoculated with 7702 (n = 8), ΔbclA (n = 8), ΔbclA/BclA (n = 8) spores or PBS (n = 6). BAL fluids were collected 6 hours later and C5a level in the supernatant determined using the Mouse Complement Component C5a DuoSet. Data shown were combined from two independent experiments, each with duplicate wells. *, p < 0.05; **, p < 0.01. ****, p < 0.0001, t test.

**Fig 5:**
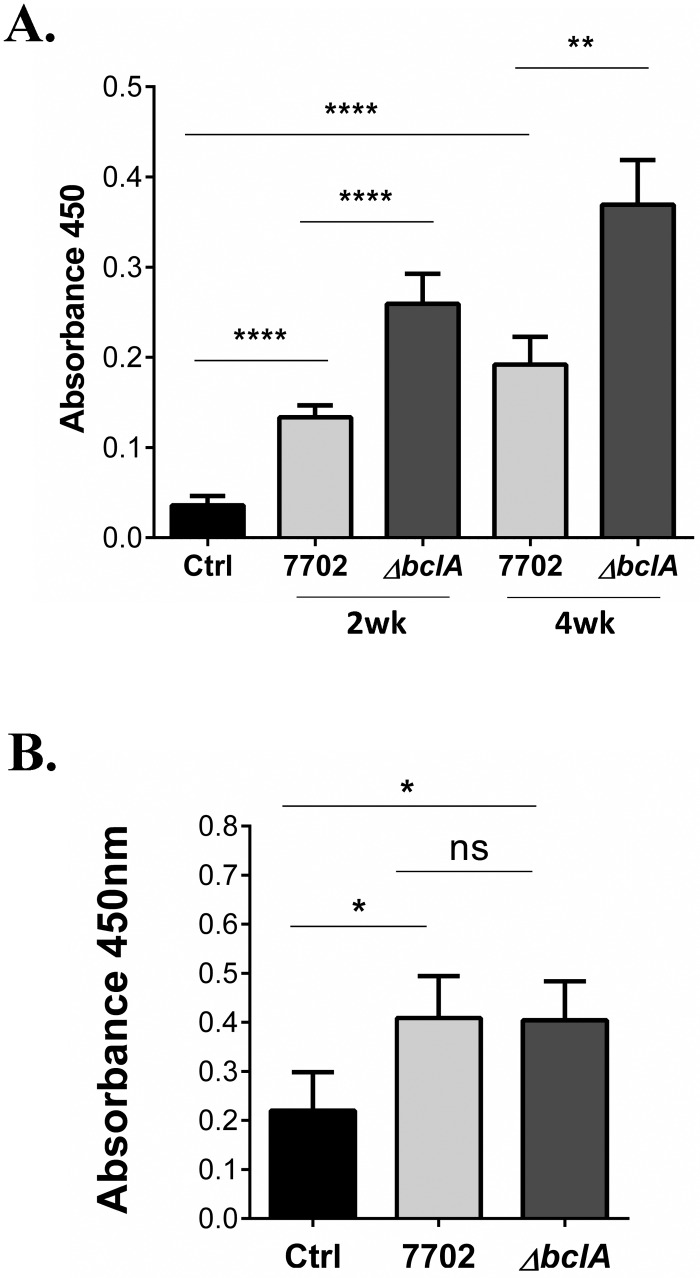
BclA inhibited antibody responses against spores. (**A**) C57BL/6 mice were i.n. inoculated with ~1×108 spores of 7702, ΔbclA or vehicle control once and blood collected at 2 weeks post inoculation (2wk), or inoculated again with the same spores and dose at 2 weeks and blood collected at 4 weeks after the initial inoculation (4wk). Anti-spore antibodies in the serum were detected using ELISA. Data shown were combined from at least three independent experiments. The mouse number for the various groups is as follows: control, n = 8; 2wk experiment, n = 30 and 29 for 7702 and ΔbclA, respectively; 4wk experiment, n = 30 and 28 for for 7702 and ΔbclA, respectively. (**B**) C3-/- mice were i.n. inoculated with vehicle control, or ~5×105 spores of 7702 or ΔbclA and blood collected at 2 weeks post inoculation. Anti-spore antibodies in the serum were detected using ELISA. Data shown were combined from at least three independent experiments, with n = 10, 24 and 21 for control, 7702 and ΔbclA, respectively. *, p < 0.05; **, p < 0.01; ****, p < 0.0001; t test.
