# Correction: *Bacillus anthracis* Spore Surface Protein BclA Mediates Complement Factor H Binding to Spores and Promotes Spore Persistence

**DOI:** 10.1371/journal.ppat.1007579

**Published:** 2019-02-08

**Authors:** Yanyu Wang, Sarah A. Jenkins, Chunfang Gu, Ankita Shree, Margarita Martinez-Moczygemba, Jennifer Herold, Marina Botto, Rick A. Wetsel, Yi Xu

The authors would like to correct Fig 1. Fig 1C contained two splice lines in the row labeled “CFH, ~155 kDa,” between lines “ΔbclA” and “ΔbclA/BclA.” Please see the complete, correct Fig 1 here.

**Fig 1:**
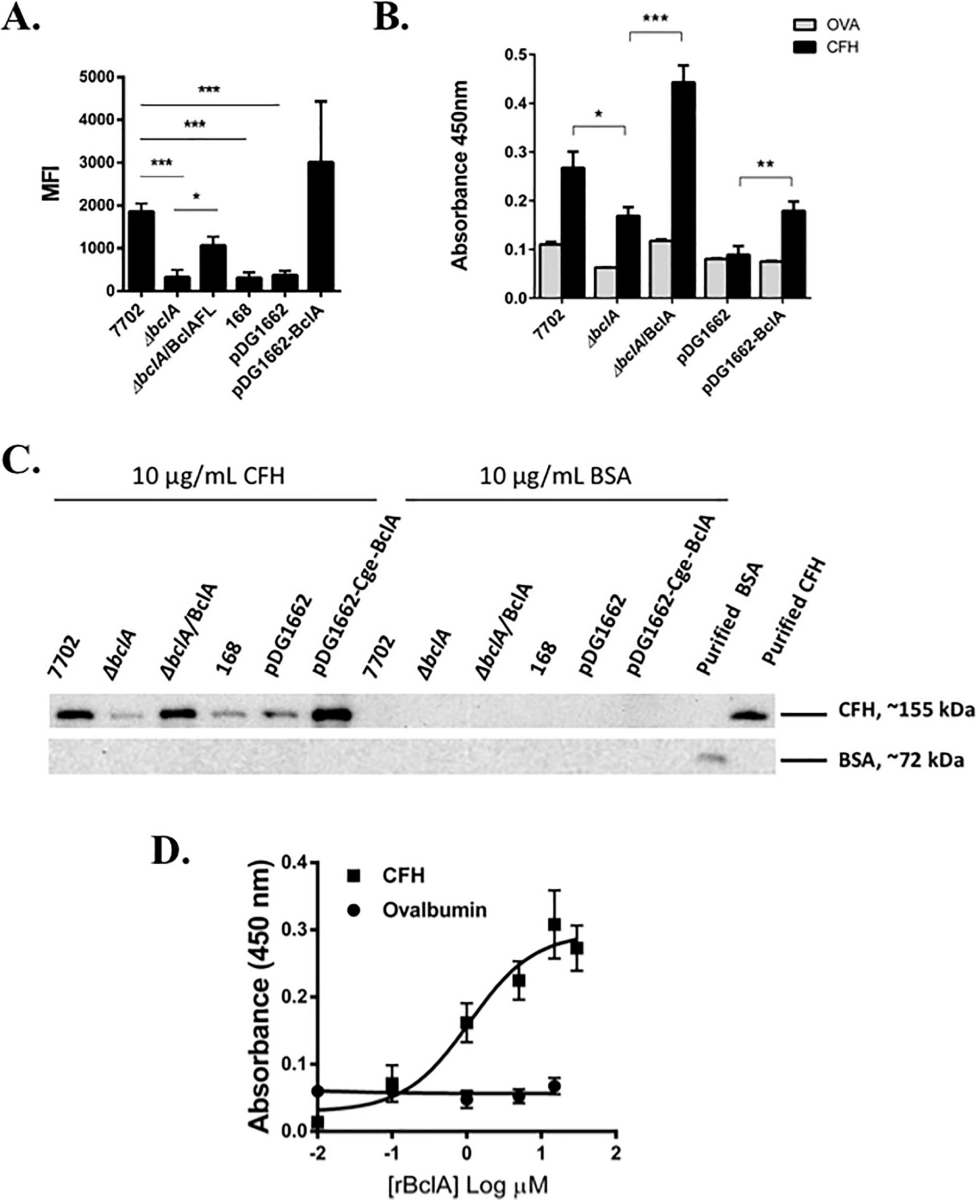
*B*. *anthracis* spore surface protein BclA mediated CFH binding to spores. Spores were incubated with purified human CFH in PBS buffer containing D-alanine. Spore-bound CFH was determined by flow cytometry (**A**), solid phase binding assay (**B**) and Western blot (**C**). Flow cytometry results were combined from at least three independent experiments. Solid phase binding assay results were combined from two independent experiments, each with duplicate wells. Western blots shown were representative of at least three independently performed experiments. (**D**) Recombinant BclA protein (rBclA) bound to immobilized human CFH in a concentration-dependent manner. Results were combined from three independent experiments. *, *p* < 0.05; **, *p* < 0.01; ***, *p* < 0.001; *t* test.

The authors confirm that these changes do not alter their findings.
