# Supplementary material for: Bacillus anthracis Spore Surface Protein BclA Mediates Complement Factor H Binding to Spores and Promotes Spore Persistence
Source: PLoS Pathog. 2016 Jun 15;12(6):e1005678. doi: 10.1371/journal.ppat.1005678 (PMC4909234; doi:10.1371/journal.ppat.1005678)
Supplement: S1 Text — (DOCX) [file ppat.1005678.s001.docx]

**S1 Text - Supporting Methods and Materials**

**Immunofluorescence microscopy and flow cytometry to confirm BclA expression on spores.** Immunoflorescence microscopy was done following a procedure descried previously (4) with modifications. Spores of *B. anthracis* 7702, *∆bclA* and *∆bclA*/BclA, and *B. subtilis* pDG1662-CgeA vector control and *B. subtilis* pDG1662-CgeA-BclA were labeled with Texas Red-X succinimidyl ester (Invitrogen), resuspended in 500 µL ice-cold sterile PBS, added to poly-L-lysine coated coverslips in 24-well plates and spun down. The cover slides were washed twice with PBS. The spores were fixed by 2% paraformaldehyde, blocked with 5% goat serum in PBS, and incubated with rabbit anti-BclA antiserum (1:1000) or pre-bleed rabbit serum (1:1000) followed by goat anti-rabbit antibodies conjugated to Alexa Fluor 488 or 594 in 2.5% fetal bovine serum. Images were taken using a DeltaVision Deconvolution microscope (GE Health) or Nikon Eclipse TS100. For flow cytometry analysis, spores were incubated with rabbit anti-BclA antiserum (1:1000) followed by secondary antibodies conjugated to Alexa Fluor 594 or secondary antibodies only. Samples were analyzed in a two laser Accuri C6 analytical flow cytometer.

**Germination assay.** The assay was performed as described (90) with slight modifications. Briefly, **s**pores of *B. anthracis* 7702, *∆bclA* and *∆bclA*/BclA were heat activated at 68 ºC for 30 min. Activated spores were washed twice with a buffer (50 mM Tris-HCl, pH 7.4, 10 mM NaCl) and resuspended in 200 µL of normal human serum (NHS), LB or a germination buffer (50 mM Tris-HCl, pH 7.4, 10 mM NaCl , 100 mM L-alanine) to reach an OD_580_ of 1.0. Spore suspensions were added to wells in the 96-well plate. The plate was placed in a Synergy H1 Multi-Mode Reader (BioTek) with the temperature set to 37°C. OD at 580 nm or 600 nm was recorded every 5 or 10 min for 60 min. The reader was set to shake the plate for 10 seconds before each reading. The experiment was performed twice, each with duplicate wells.

**Histology.** Lungs were collected from C57BL/6 mice two weeks post i.n. inoculation of 1$\times$10^8^ 7702 or *∆bclA*  spores, and fixed in 10% formalin. The sections (5 μm) were prepared and hematoxylin and eosin staining of lung sections performed by North Bay Histology Lab (Novato, CA).
