# Supplementary material for: Bacillus anthracis Spore Surface Protein BclA Mediates Complement Factor H Binding to Spores and Promotes Spore Persistence
Source: PLoS Pathog. 2016 Jun 15;12(6):e1005678. doi: 10.1371/journal.ppat.1005678 (PMC4909234; doi:10.1371/journal.ppat.1005678)
Supplement: S1 Table — (DOCX) [file ppat.1005678.s002.docx]

**S1 Table. Strains and plasmids used in this study.**

| Strain or plasmid | Relevant characteristic(s) | Reference or source |
| --- | --- | --- |
| *B. anthracis* |  |  |
| 7702 | pXO1^+^, pXO2^−^ | [4] |
| *∆bclA* | A 7702 mutant strain with codons 27-382 of the *bclA* gene deleted. | [28] |
| *∆bclA*/BclA | *∆bclA* strain containing plasmid pUTE583-BclA | This study |
| *B. subtilis* |  |  |

| 168 | *trpC2* | *Bacillus* genetic Stock Center |
| --- | --- | --- |
| PY79 |  | [90] |

| 168/pDG1662 | *B. subtilis* 168 containing the integration vector pDG1662, integrated at the *amyE* locus | This study |
| --- | --- | --- |
| 168/pDG1662-BclA | *B. subtilis* 168 containing pDG16662-BclA integrated at the *amyE* locus | This study |
| *E. coli* |  |  |
| BL21/pBAD-rBclA | BL21 rosetta (Novagen) containing pBAD-rBclA | This study |
| Plasmids |  |  |
| pUTE583 | A low copy number *E. coli* – *B. anthracis* shuttle vector; Cm^r^ in *E. coli*, Em^r^ in *B. anthracis* | [81] |
| pUTE583-BclA | pUTE583 carrying a DNA fragment containing the *B. anthracis bclA* gene and its ~1kb upstream sequence | This study |
| pDG1662 | A chromosomal integration vector of *B. subtilis, amyE’*, *cat*, *bla*, *spc* | *Bacillus* genetic Stock Center, [86] |
| pDG1662-BclA | pDG1662 carrying a DNA fragment encoding 39-400 amino acids of *B. anthracis* BclA fused to the C-terminus of *B. subtilis* CgeA. | This study |
