# Supplementary figures and images for: Bacillus anthracis Spore Surface Protein BclA Mediates Complement Factor H Binding to Spores and Promotes Spore Persistence
Source: PLoS Pathog. 2016 Jun 15;12(6):e1005678. doi: 10.1371/journal.ppat.1005678 (PMC4909234; doi:10.1371/journal.ppat.1005678)

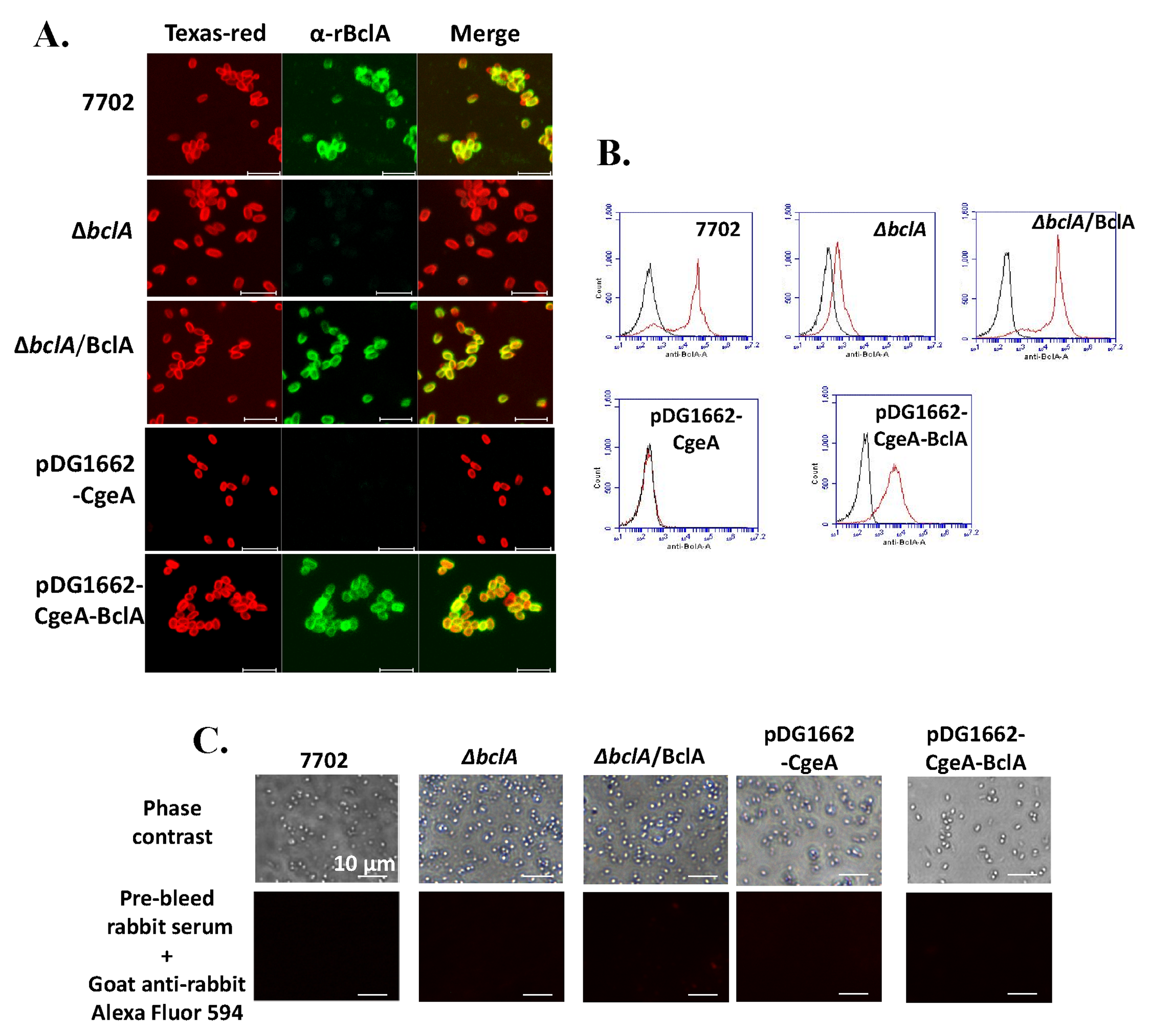

Supplement: S1 Fig — Immunofluorescence microscopy and flow cytometry was performed as described in Supporting Methods and Materials. A. Imunofluorescence microscopy of spores of B. anthracis 7702, ΔbclA, ΔbclA/BclA, and B. subtilis pDG1662-CgeA vector only and B. subtilis pDG1662-CgeA-BclA. Spores were labeled with Texas Red to visualize spores. They were then incubated with rabbit anti-BclA antiserum and goat anti-rabbit antibodies conjugated to Alexa Fluor 488. B. Flow cytometry analysis. Spores were incubated with anti-BclA antiserum and secondary antibodies conjugated to Alexa Fluor 594, or with secondary antibodies only. C. Spores were incubated with rabbit pre-bleed serum and secondary antibodies conjugated to Alexa Fluor 594. The spores were then examined by phase contrast and fluorescence microscopy. (TIF) [file ppat.1005678.s003.tif]

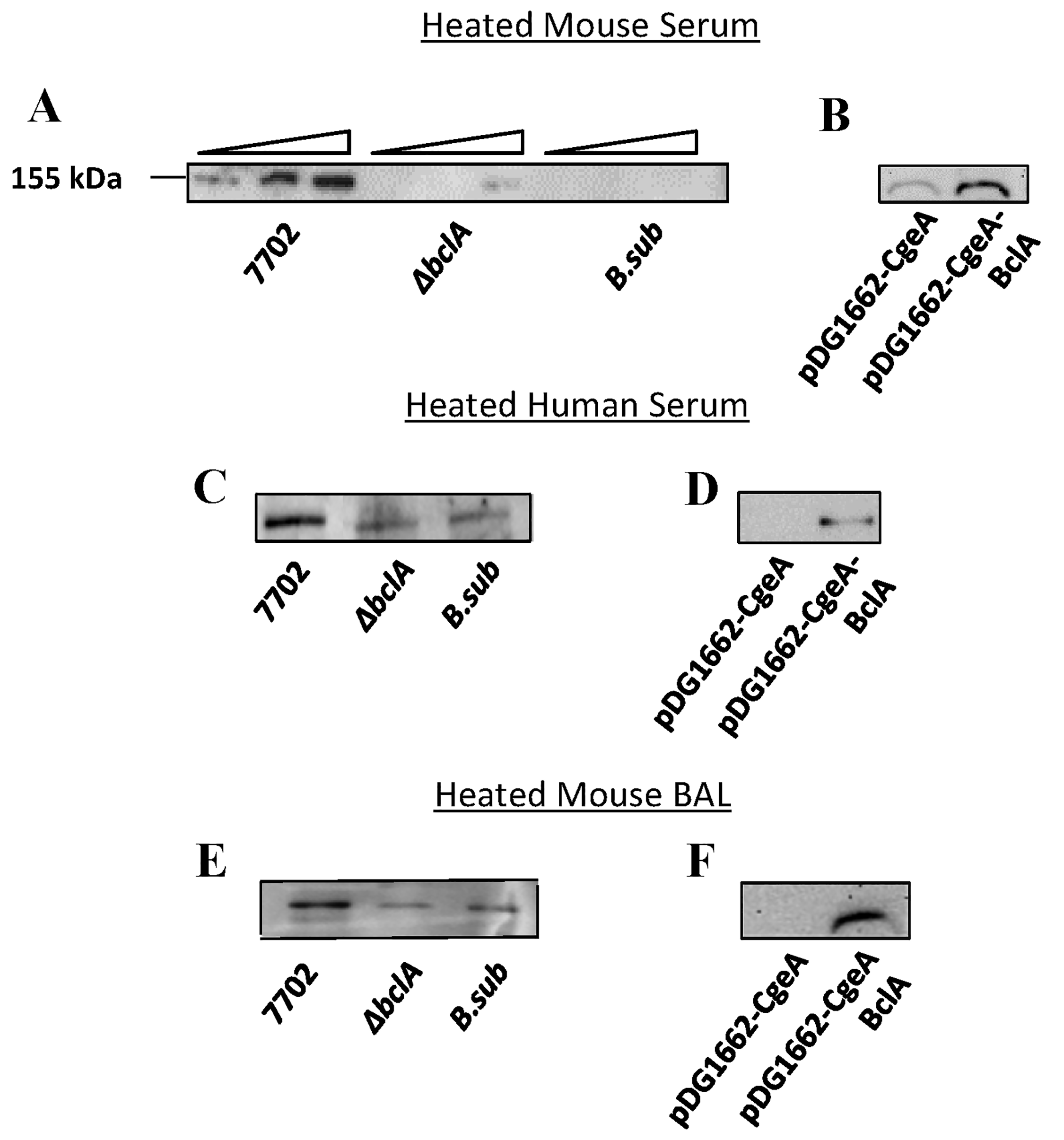

Supplement: S2 Fig — Spores of B. anthracis 7702 and ΔbclA, and B. subtilis (B. sub), B. subtilis carrying pDG1662-CgeA vector control (pDG1662) and pDG1662-CgeA-BclA (pDG1662-BclA) were incubated in 10% heat-inactivated mouse serum (A and B), 10% heat-inactivated human serum (C and D) or heat-inactivated BAL fluid (E and F) at 37°C for 30 min. Spore-bound CFH was detected using pull down assays as described in the Methods and Materials section. Different amounts of spores (~5×10^6^, 5×10^7^ and 5×10^8^ spores) were used for the experiment in panel A, and ~ 5×10^7^ spores were used for experiments in panels B—F. Data shown were from representative experiments. (TIF) [file ppat.1005678.s004.tif]

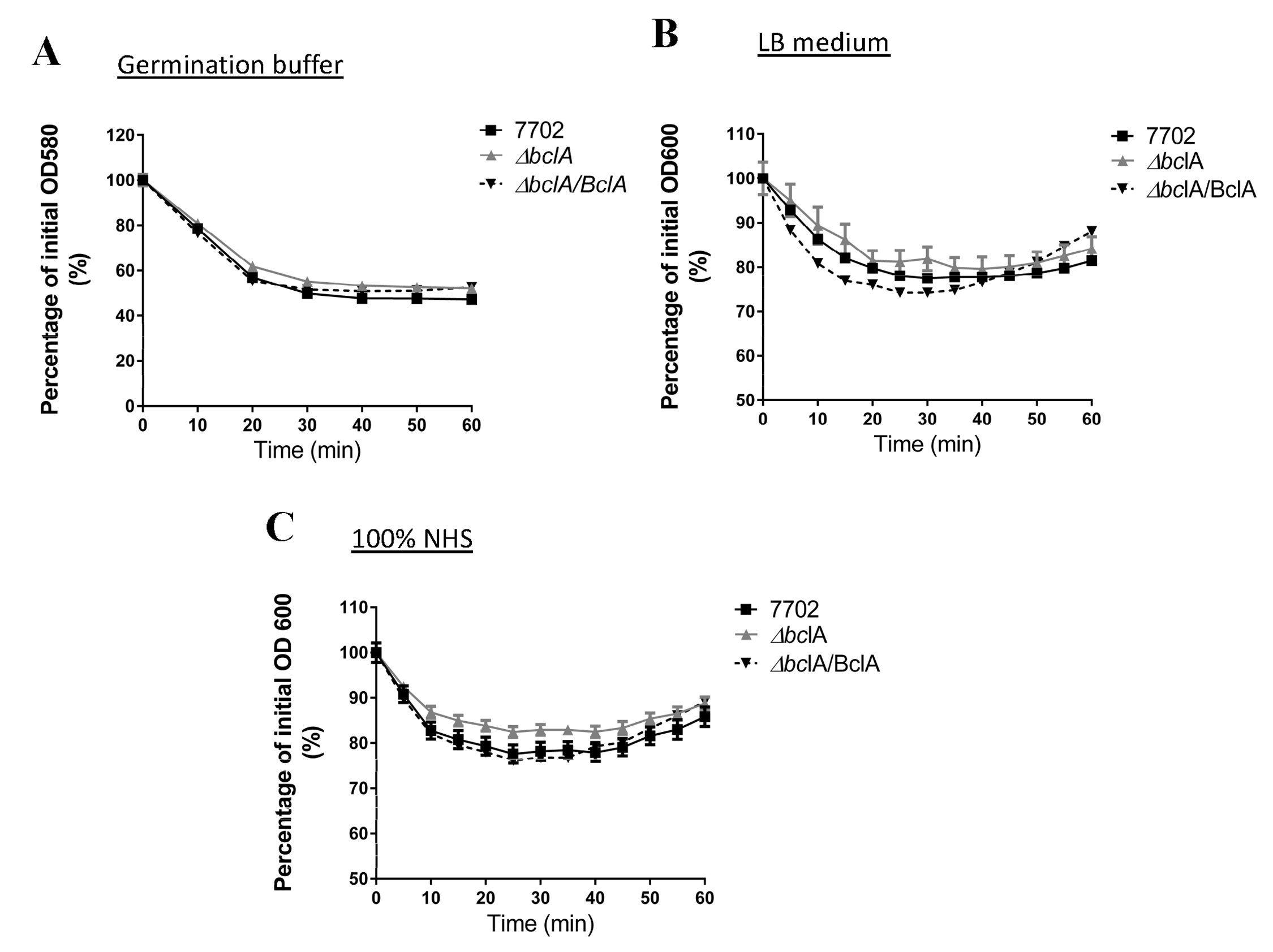

Supplement: S3 Fig — Spores were heat activated at 68°C for 30 min and resuspended in a germination buffer (50 mM Tris-HCl, pH 7.4, 10 mM NaCl, 100 mM L-alanine) (A), LB (B) or NHS (C) to reach OD580 of 1.0. Kinetic readings were performed every 5 or 10 min at 37°C for 60 min using a Synergy H1 Multi-Mode Reader. The experiment was performed twice, each with duplicate wells. Data was normalized to OD at time zero. (TIF) [file ppat.1005678.s005.tif]

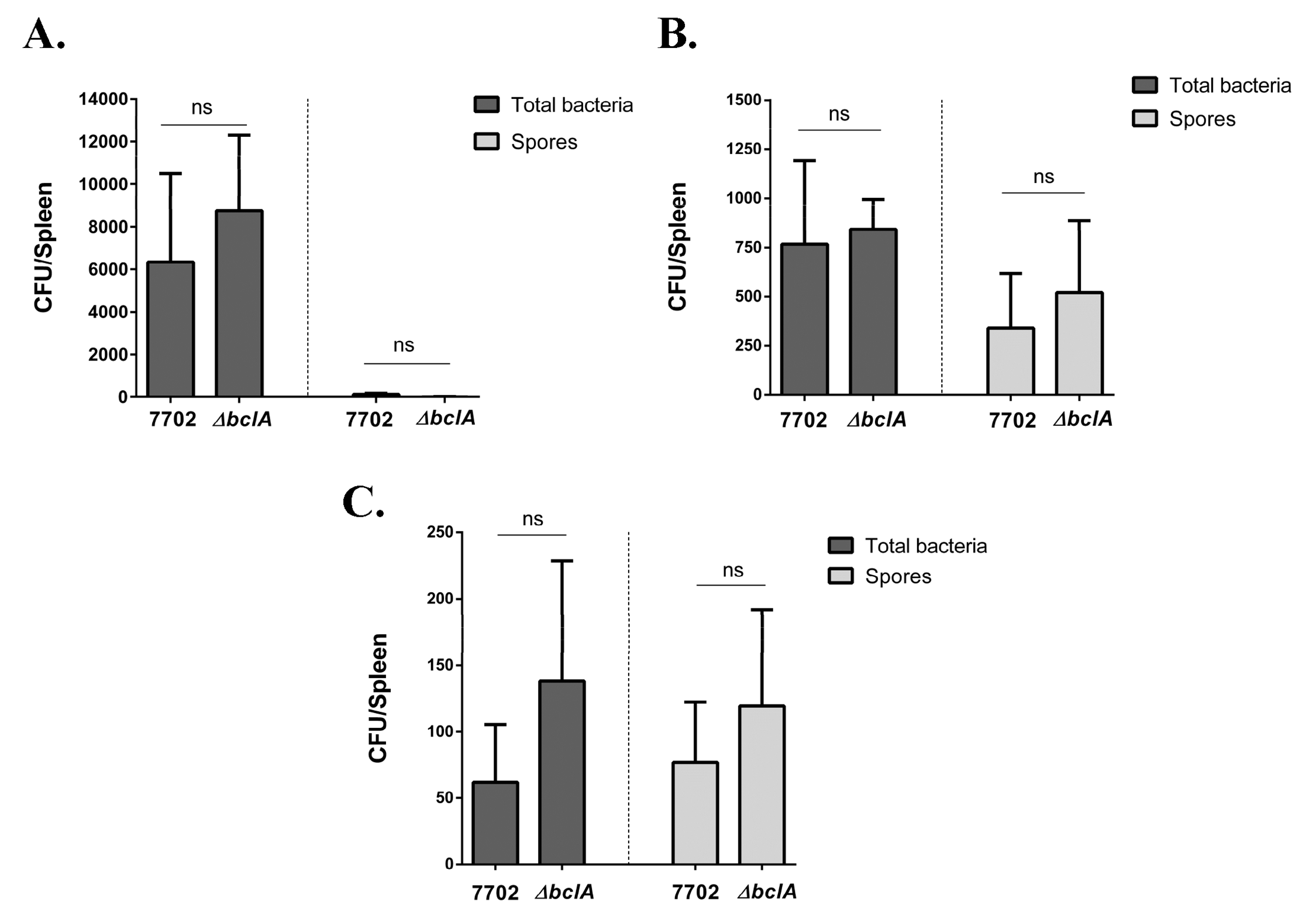

Supplement: S4 Fig — C57BL/6 (A) and C1q-/- (B) mice were i.n. inoculated with ~1×10^8^ spores per mouse. C3-/- (C) mice were i.n. inoculated with ~ 5×10^5^ spores per mouse. Spleens were collected at 2 weeks post inoculation and homogenized in 1ml sterile PBS containing 2.5 mM D-alanine. The homogenates were either plated directly to determine the total viable bacterial counts or heated at 68°C for 1 hr and dilution plated to determine spore counts. Data shown were combined from at least two independent experiments. C57BL/6, n = 12 and 7 for 7702 and ΔbclA, respectively; C1q-/-, n = 6 and 5 for 7702 and ΔbclA, respectively; C3-/-, n = 6 and 5 for 7702 and ΔbclA, respectively. (TIF) [file ppat.1005678.s006.tif]

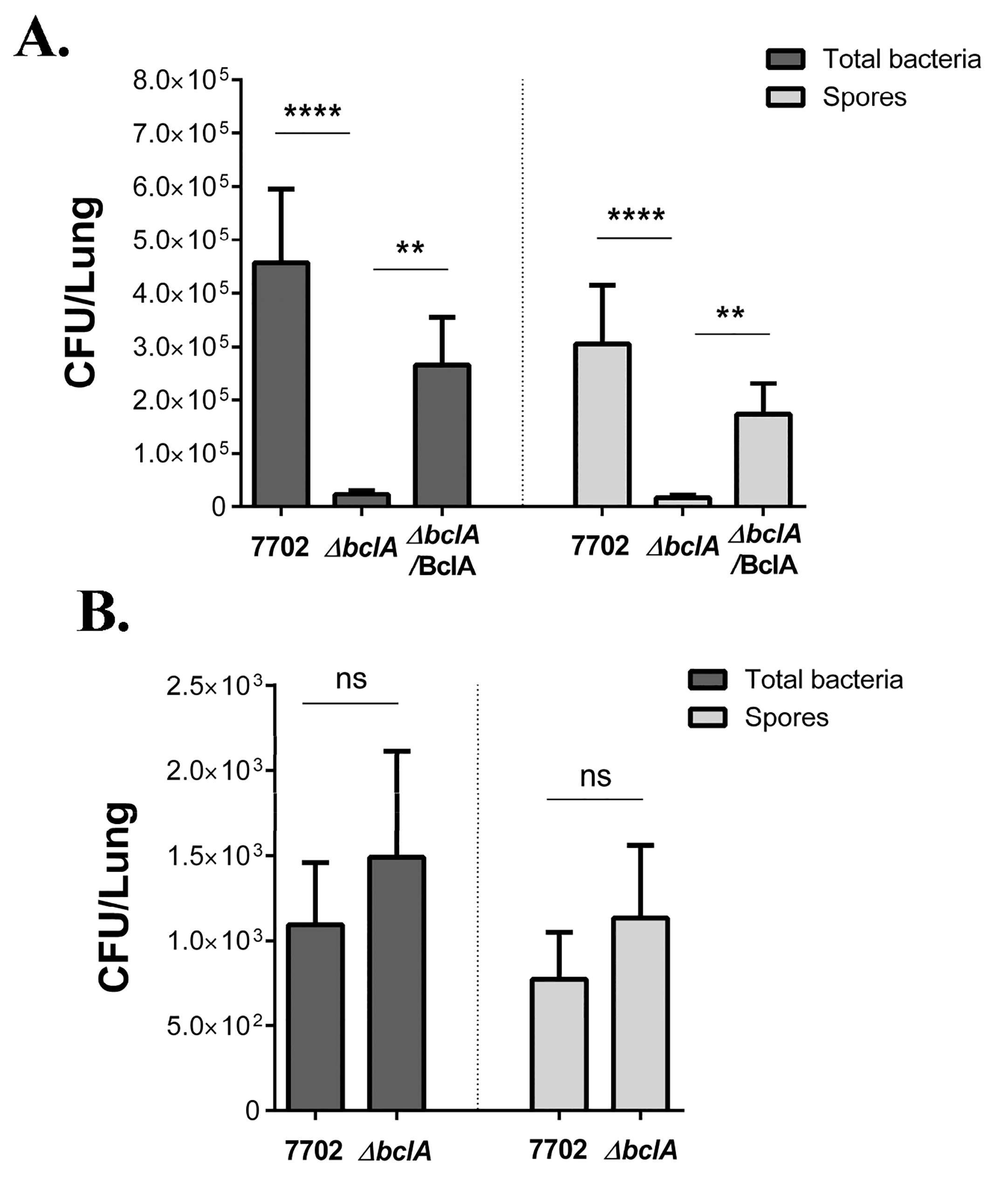

Supplement: S5 Fig — This was performed as described in the legend for Fig 4 except that lungs were collected at 4 weeks post inoculation. C57BL/6 (A) and C3-/- (B) mice were i.n. inoculated with sub-lethal doses of spores of 7702, ΔbclA or ΔbclA/BclA. Bacterial and spore load in the lungs at 4 weeks post inoculation was determined. Data shown were combined from at least two independent experiments. C57BL/6 mice, n = 14, 15, and 14 for 7702, ΔbclA and ΔbclA/BclA, respectively; C3-/- mice, n = 9 and 7 for 770 and ΔbclA, respectively. **, p < 0.01; ****, p < 0.0001; t test. (TIF) [file ppat.1005678.s007.tif]

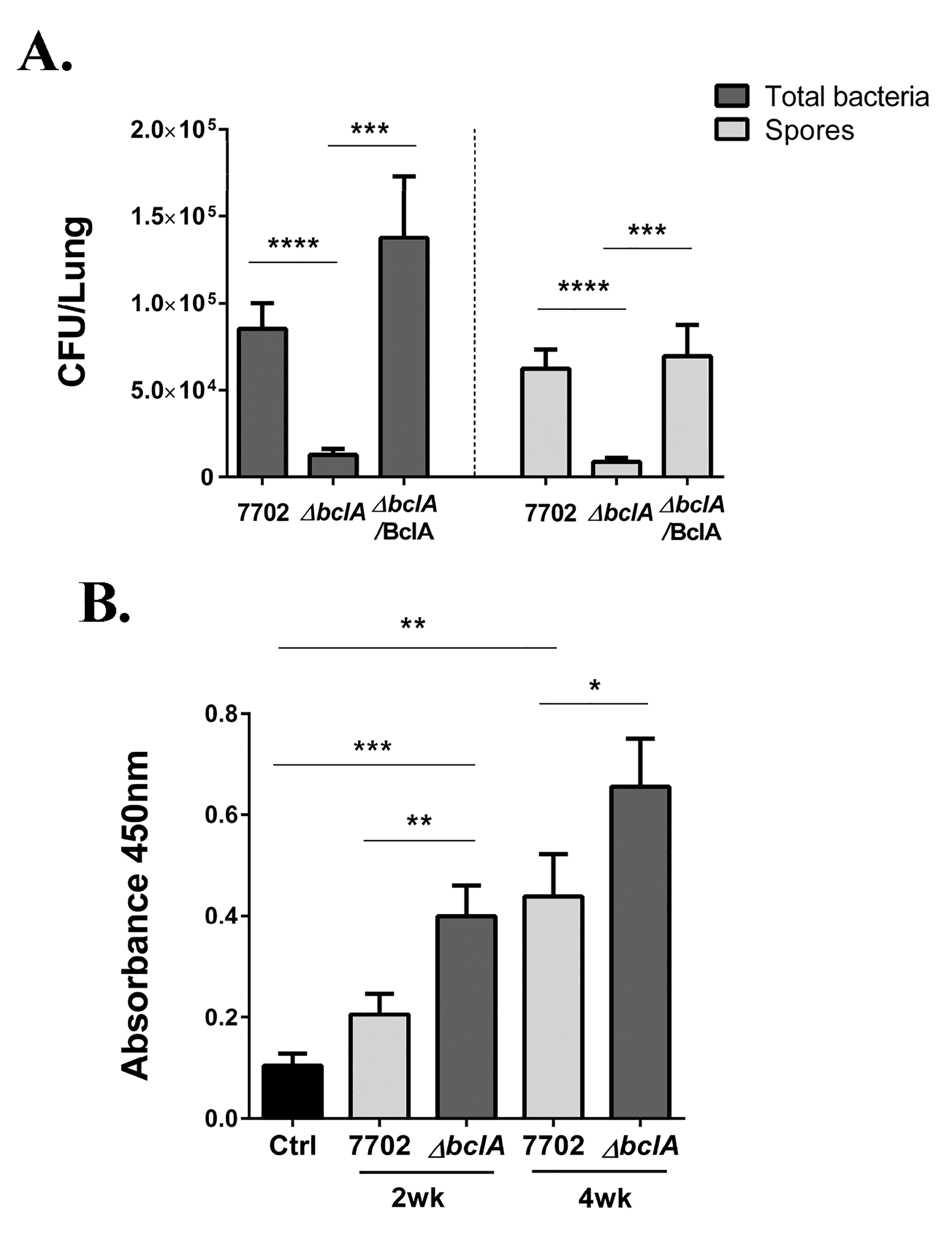

Supplement: S6 Fig — (A) C1q-/- mice were i.n. inoculated with ~ 1×10^8^ spores per mouse of 7702 (n = 7), ΔbclA (n = 9) or ΔbclA/BclA (n = 5). Lungs were collected at 2 weeks post inoculation, and total viable bacteria and spore counts determined. Data shown were combined from two independent experiments. (B) C1q-/- mice were i.n. inoculated with ~ 1×10^8^ spores per mouse of 7702, ΔbclA or vehicle control once (2wk) or twice (4wk). Blood was collected 2 weeks after the last inoculation. Antibody titers in the serum were measured using ELISA with spore protein extracts as antigens. Data shown were combined from at least two independent experiments. Ctrl, n = 6; 7702-2wk, n = 17; ΔbclA (2wk), n = 12; 7702-4wk, n = 17; ΔbclA (2wk), n = 12. *, p < 0.05; **, p < 0.01; ***, p < 0.001; ****, p < 0.0001; t test. (TIF) [file ppat.1005678.s008.tif]

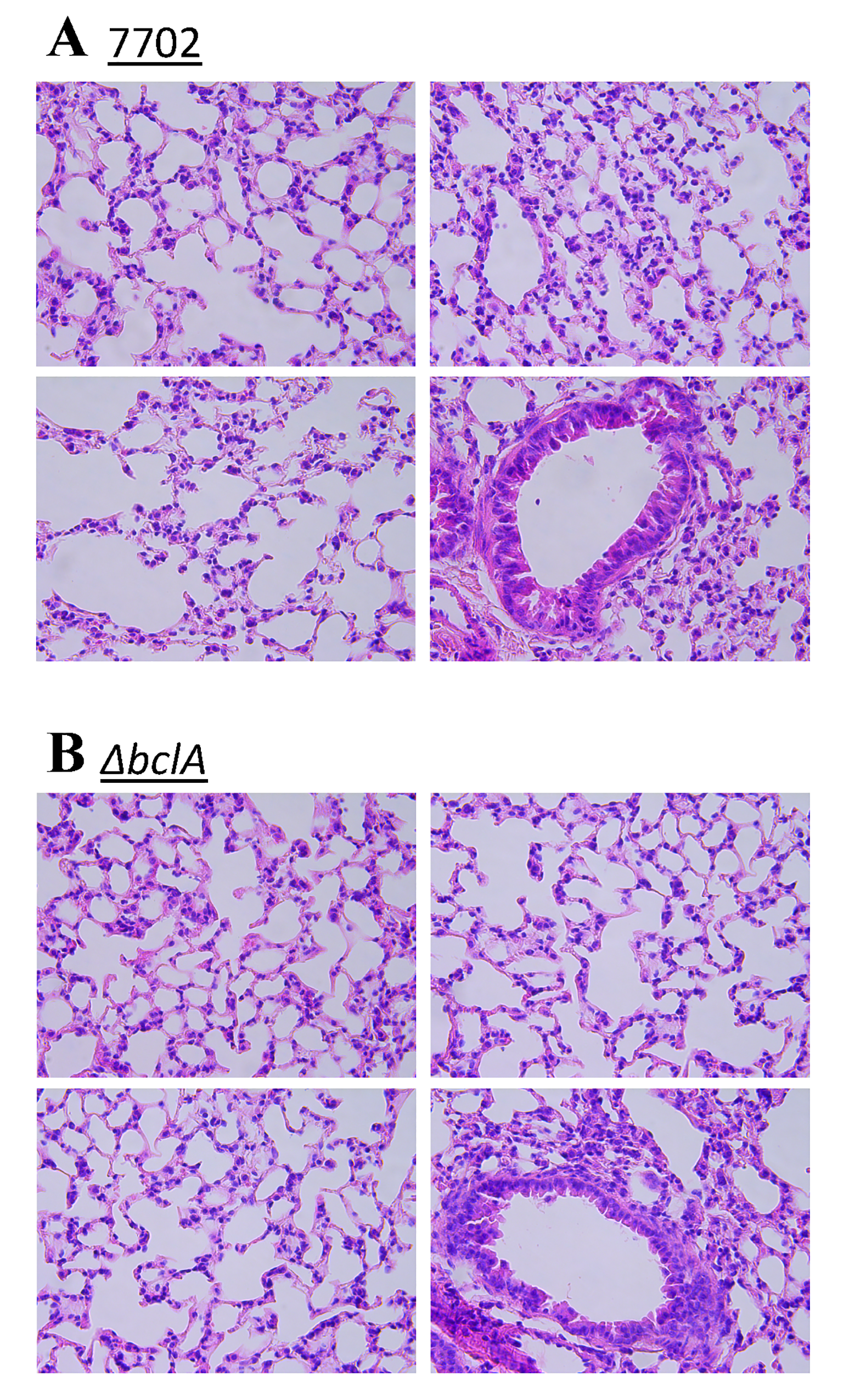

Supplement: S7 Fig — C57BL/6 mice were i.n. inoculated with 1×10^8^ 7702 or ΔbclA spores and lungs collected at 2 weeks post inoculation. Representative images of lung sections from 7702 (A) and ΔbclA (B)-inoculated mice (n = 2/group) are shown. (TIF) [file ppat.1005678.s009.tif]
